# Postnatal heart development is coordinated by multi-lineage Wnt and BMP signaling

**DOI:** 10.1242/dev.205448

**Published:** 2026-10-06

**Authors:** Hui An, Hao Hu, Saijing Chen, Tingting Chen, Yafei Hu, Tongtong Yu, Anlan Wang, Shengwei Jin, Ming Li

**Affiliations:** ^1^ Department of Anesthesia and Critical Care, The Second Affiliated Hospital and Yuying Children's Hospital of Wenzhou Medical University; Key Laboratory of Pediatric Anesthesiology (Wenzhou Medical University), Ministry of Education; Key Laboratory of Precision Anesthesiology of Zhejiang Province, Wenzhou Medical University, Zhejiang 325027, China; ^2^ School of Basic Medical Science, Wenzhou Medical University, Wenzhou, Zhejiang 325035, China

**Keywords:** Postnatal heart development, Cardiomyocyte enlargement, Wnt and BMP signaling, β-catenin, Paracrine BMP4, Multi-lineage coordination

## Abstract

Early postnatal heart growth requires a transition from cardiomyocyte proliferation to enlargement and binucleation. Using morphometric, transcriptomic, signaling and single-cell analyses in neonatal mice, we show that this transition occurs predominantly between postnatal days 3.5 and 4.5 (P3.5-P4.5), accompanied by declining β-catenin abundance and increased SMAD1/5/9 phosphorylation. Pharmacological disruption of β-catenin/CBP signaling or Ctnnb1 knockdown promotes cardiomyocyte enlargement, whereas β-catenin stabilization by CHIR-99021 enhances cell-cycle activity and estimated ventricular cardiomyocyte number while limiting enlargement. Transcriptomic analysis reveals a Sox2/Zic1/Olig2 module enriched in early neural/glial cells, suggesting the onset of postnatal cardiac nervous system development. Wnt pathway components are active in endothelial, mesenchymal and neural populations during the early postnatal window, whereas BMP signaling peaks in cardiomyocytes at P6. These findings reveal a multi-lineage interplay between Wnt and BMP signaling that balances cardiomyocyte number expansion versus enlargement during early postnatal heart development.

## Teaser

**Summary:** Between postnatal days 3.5 and 4.5, mouse heart growth shifts from cardiomyocyte number expansion toward cardiomyocyte enlargement, alongside declining β-catenin abundance and increasing BMP-related signaling across multiple cardiac cell populations.

## INTRODUCTION

Cardiovascular diseases remain the leading cause of mortality worldwide, a burden that is further compounded by the limited regenerative capacity of the adult mammalian heart (Derks et al., 2025; Li et al., 1996). In contrast, lower vertebrates such as zebrafish regenerate myocardium primarily through proliferation of pre-existing cardiomyocytes (Weinberger and Riley, 2024), and neonatal mice retain a transient regenerative capacity during the earliest postnatal period (Weinberger and Riley, 2024; Porrello et al., 2011; Naqvi et al., 2014). This short developmental window coincides with rapid changes in cardiomyocyte cell-cycle activity, nuclearity, size, metabolism and tissue growth (Derks et al., 2025; Yan et al., 2021). Understanding how the neonatal heart shifts from a cell-cycle-associated growth state toward postnatal cardiomyocyte enlargement is therefore important for defining mechanisms that may be leveraged to improve cardiac repair.

Early postnatal cardiac growth is a dynamic and non-uniform process. During this period, cardiomyocytes progressively reduce proliferative activity, undergo binucleation and increase in size, while the heart continues to expand rapidly. In the postnatal mammalian heart, growth is achieved predominantly through physiological cardiomyocyte enlargement because most cardiomyocytes exit the cell cycle and transition from number expansion toward cell enlargement growth (Zhao et al., 2025; Swift et al., 2023; Bishop et al., 2021; Velayutham et al., 2020). Such cell enlargement growth can be physiological, accompanying normal development or exercise, or pathological, triggered by pressure overload or mutations in sarcomeric proteins (Wulfsohn et al., 2004; Li et al., 1996). These growth programs are regulated by interconnected signaling pathways that reprogram gene expression to direct cardiomyocyte growth and tissue remodeling (DeLaughter et al., 2016; Horitani and Shiojima, 2024; Weinberger and Riley, 2024). However, the timing and signaling mechanisms that coordinate the early postnatal balance between cardiomyocyte number expansion and cardiomyocyte enlargement remain incompletely understood.

Wnt/β-catenin signaling is a central regulator of embryonic development, cell fate determination and tissue growth, but its function in the heart is highly context dependent (Horitani and Shiojima, 2024; Xia et al., 2025; Ozhan and Weidinger, 2015; Zelarayan et al., 2007; Wei et al., 2008). Studies in both zebrafish and mammalian models have demonstrated that Wnt and β-catenin signaling exerts biphasic or even opposing effects at different stages of cardiac development and during injury responses (Ozhan and Weidinger, 2015; Tzahor, 2007). In adult cardiac injury or stress models, inhibition of Wnt/β-catenin signaling has been associated with improved tissue repair and functional recovery, whereas β-catenin stabilization can impair adaptive hypertrophic remodeling under hypertensive stress (Bergmann, 2010; Chen et al., 2006b; Baurand et al., 2007). These observations raise the possibility that β-catenin-associated signaling may also influence the balance between cardiomyocyte proliferation-associated activity and cardiomyocyte enlargement during early postnatal development. However, its role during this short developmental window, when cardiomyocytes reduce cell-cycle activity and initiate physiological growth, remains insufficiently defined.

BMP/SMAD signaling is another major developmental pathway involved in cardiac lineage specification, tissue remodeling and cardiomyocyte growth. BMP/SMAD signaling modulates neurotrophin-mediated axonal growth and regulates the differentiation of neural progenitors (Zhong and Zou, 2014; Gambaro et al., 2006). During cardiogenesis, BMP signaling promotes cardiac transcriptional programs, including regulators such as Gata4, Nkx2.5 and Mef2c, and can induce cardiomyocyte differentiation from precardiac mesoderm in embryonic models (Tirosh-Finkel et al., 2010). Beyond embryonic development, BMP signaling, particularly through BMP2 and BMP4, has also been implicated in postnatal cardiomyocyte enlargement growth (Shahid et al., 2016). Wnt and BMP pathways interact in a context-dependent manner across developmental systems; for example, enhanced Wnt signaling can inhibit BMP-mediated differentiation in C2C12 myoblasts, indicating that Wnt-BMP crosstalk can shape lineage and growth-related outcomes (Nakashima et al., 2005). Whether Wnt/β-catenin- and BMP/SMAD-related programs undergo coordinated temporal remodeling during early postnatal cardiac growth, and whether this remodeling is linked to the transition from cell cycle-associated activity to cardiomyocyte enlargement, remains unclear. A further challenge is that whole-heart transcriptomic changes reflect contributions from multiple cardiac cell types, including cardiomyocytes, endothelial cells, epicardial cells, fibroblast-like mesenchymal cells, neural and glial-like populations, and other resident cells. Thus, resolving the cellular context of Wnt- and BMP-associated transcriptional programs is essential for interpreting how these pathways contribute to early postnatal cardiac remodeling.

Here, we define the morphometric, transcriptomic, signaling and cellular features of early postnatal heart growth in mice. We identify the P3.5-to-P4.5 interval as a key developmental window during which cardiomyocyte enlargement and binucleation coincide with a transcriptional shift from cell cycle-associated programs toward muscle growth-associated remodeling. We further show that β-catenin abundance declines, while SMAD1/5/9 phosphorylation increases during this transition. Pharmacological disruption or knockdown of β-catenin-associated signaling enhances cardiomyocyte enlargement, whereas β-catenin stabilization promotes cardiomyocyte cell cycle-associated activity and increases estimated ventricular cardiomyocyte number while limiting cell-volume expansion. Finally, single-cell transcriptomic analysis places these changes within a multi-cell-type framework, showing that Sox, Zic and Olig-containing developmental modules are enriched primarily in early neural and glial-like cells, whereas cardiomyocytes follow a distinct trajectory marked by declining cell cycle activity and increasing structural and growth-associated programs. Together, these findings support a model in which β-catenin-associated signaling contributes to the balance between cardiomyocyte number expansion and cardiomyocyte growth during early postnatal heart development.

## RESULTS

### Postnatal cardiac growth is accompanied by cardiomyocyte enlargement and binucleation

During the first 9 postnatal days, mouse body weight increased progressively, together with parallel increases in heart and ventricular weights. In contrast, heart weight-to-body weight ratio (HW/BW) and ventricular weight-to-body weight ratio (VW/BW) showed a transient elevation around P4.5, indicating a stage-dependent change in relative cardiac growth during early postnatal development (Fig. 1A-E). We next examined cardiomyocyte nuclearity and morphology from P1.5 to P5.5 using isolated ventricular cardiomyocytes (Fig. 1F). At P1.5 and P2.5, cardiomyocytes were predominantly single nucleated, whereas the proportion of double-nucleated cardiomyocytes increased from P3.5 onward and accounted for approximately one-third of the total cardiomyocyte population by P5.5 (Fig. 1G). Morphometric analysis showed progressive increases in cardiomyocyte length, width and estimated volume in both single-nucleated and double-nucleated cardiomyocytes (Fig. 1H-M). The increase in estimated cardiomyocyte volume was most pronounced between P3.5 and P4.5, temporally coinciding with the peak in HW/BW and VW/BW ratios (Fig. 1N,O). Estimated ventricular cardiomyocyte number increased modestly from P1.5 to P2.5 but remained largely unchanged thereafter through P5.5 (Fig. 1P). Together, these data indicate that early postnatal cardiac growth, particularly during the P3.5-to-P4.5 interval, is associated predominantly with cardiomyocyte enlargement and increasing binucleation rather than a sustained increase in ventricular cardiomyocyte number.

**Fig. 1.**
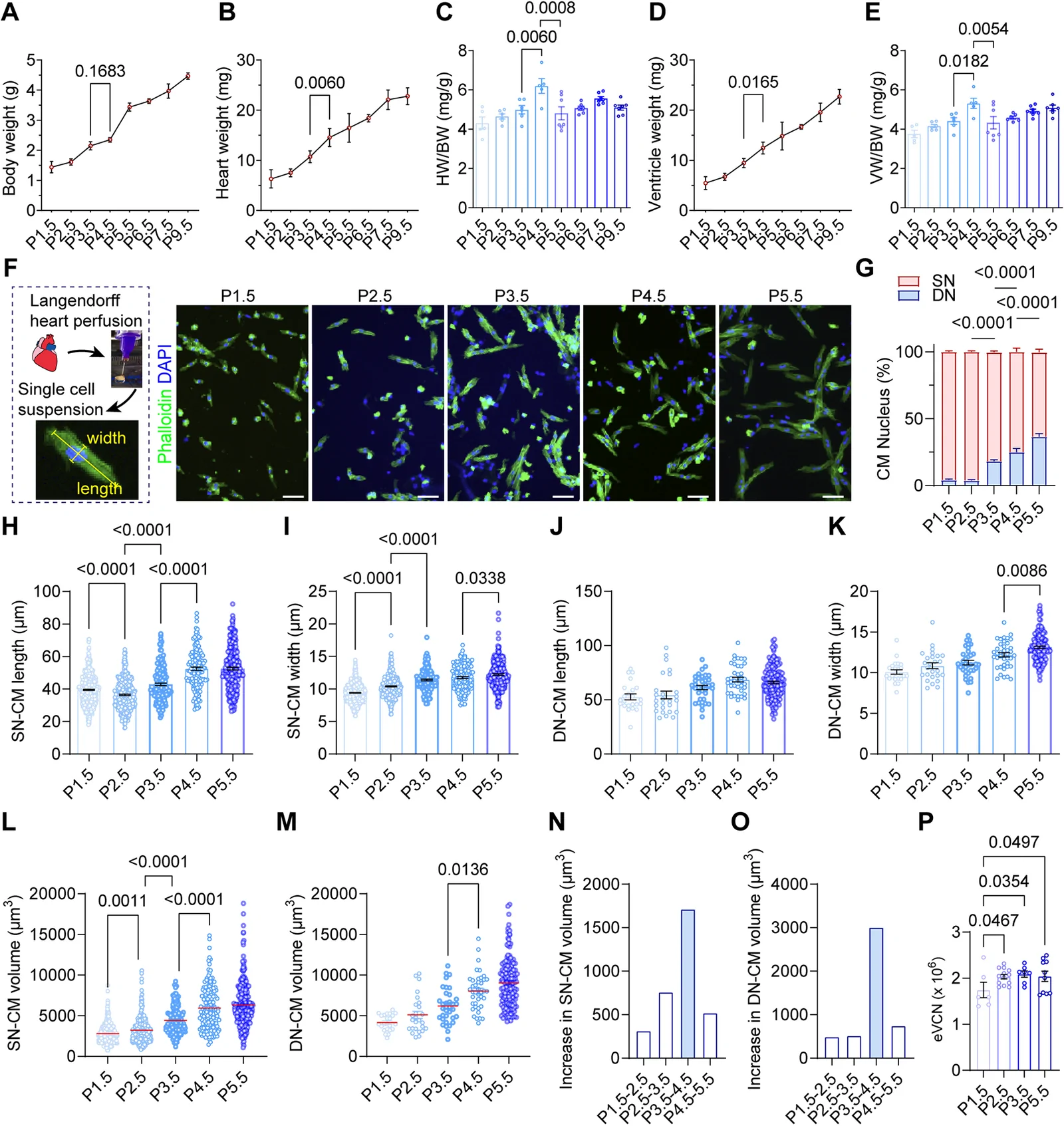
**Postnatal cardiac growth is accompanied by cardiomyocyte enlargement and binucleation in mice.** (A) Body weight of mice from postnatal day 1.5 (P1.5) to P9.5 (P1.5, *n*=5; P2.5, *n*=6; P3.5, *n*=6; P4.5, *n*=5; P5.5, *n*=7; P6.5, *n*=6; P7.5, *n*=7; P9.5, *n*=7). (B,C) Heart weight (B) and heart weight-to-body weight ratio (HW/BW, C) (same *n* values as in A). (D,E) Ventricular weight (D) and ventricular weight-to-body weight ratio (VW/BW, E) from P1.5 to P9.5 (same *n* values as in A). (F) Schematic workflow of Langendorff-based cardiomyocyte isolation and representative fluorescence images of isolated cardiomyocytes from P1.5 to P5.5 mice. Phalloidin-FITC (green); DAPI (blue). Scale bars: 50 μm. (G) Quantification of single-nucleated (SN) and double-nucleated (DN) cardiomyocytes (CM) from P1.5 to P5.5. A total of 190-600 cardiomyocytes were analyzed per time point from three biological replicates. (H-M) Morphometric analysis: SN cell length (H), width (I) and volume (L); DN cell length (J), width (K) and volume (M). Data are individual cell measurements from *n*=3 hearts per time point (each dot represents data from one cardiomyocyte). (N,O) Increase in cell volume between adjacent stages for SN-CMs (N) and DN-CMs (O). (P) Estimated ventricular cardiomyocyte number (eVCN) (P1.5, *n*=6; P2.5, *n*=13; P3.5, *n*=7; P5.5, *n*=10). Data are presented as mean±s.e.m. Statistical significance was assessed by one-way ANOVA for A-E and H-P, and by two-way ANOVA followed by Šídák's multiple-comparisons test for G. *P*<0.05 was considered statistically significant.

### Early postnatal heart development is associated with stage-specific transcriptional programs related to the cell cycle and muscle growth

To define the transcriptional changes accompanying early postnatal cardiac growth, we performed whole-heart RNA sequencing at P0.5, P2.5, P3.5 and P4.5. Principal component analysis revealed a clear developmental trajectory across these stages, with P0.5 hearts separating from later postnatal stages and P4.5 hearts diverging from P2.5 and P3.5 hearts along the principal components (Fig. 2A). Differential expression analysis between consecutive stages further demonstrated dynamic transcriptional remodeling during this early postnatal period (Fig. 2B). Comparison of P2.5 with P0.5 hearts showed that genes upregulated at P2.5 were strongly enriched for Gene Ontology biological processes related to DNA replication, mitotic cell cycle progression, chromosome segregation and cell-cycle phase transition (Fig. 2C). Consistently, GSEA showed positive enrichment of Hallmark E2F Targets and Hallmark G2 M Checkpoint gene sets in P2.5 hearts relative to P0.5 hearts (Fig. 2D). These findings indicate that the P0.5-to-P2.5 transition is associated with enhanced cell cycle-related transcriptional activity in the neonatal heart. In contrast, genes downregulated at P2.5 were enriched for processes including muscle cell differentiation, organelle organization and cellular component disassembly, suggesting a reciprocal shift in transcriptional programs during this early developmental window (Fig. 2E). Given the presence of multiple Wnt pathway-associated genes among the differentially expressed genes, we further examined selected Wnt-related regulators during the early P0.5-to-P2.5 transition. This focused analysis showed that several Wnt-associated genes, including pathway ligands, modulators and transcriptional regulators, were upregulated together with cell-cycle and DNA replication-related genes in P2.5 hearts compared with P0.5 hearts (Fig. 2B and Fig. S1A). These findings suggest that Wnt-related transcriptional remodeling occurs in parallel with enhanced cell cycle-associated gene expression during this early postnatal window.

**Fig. 2.**
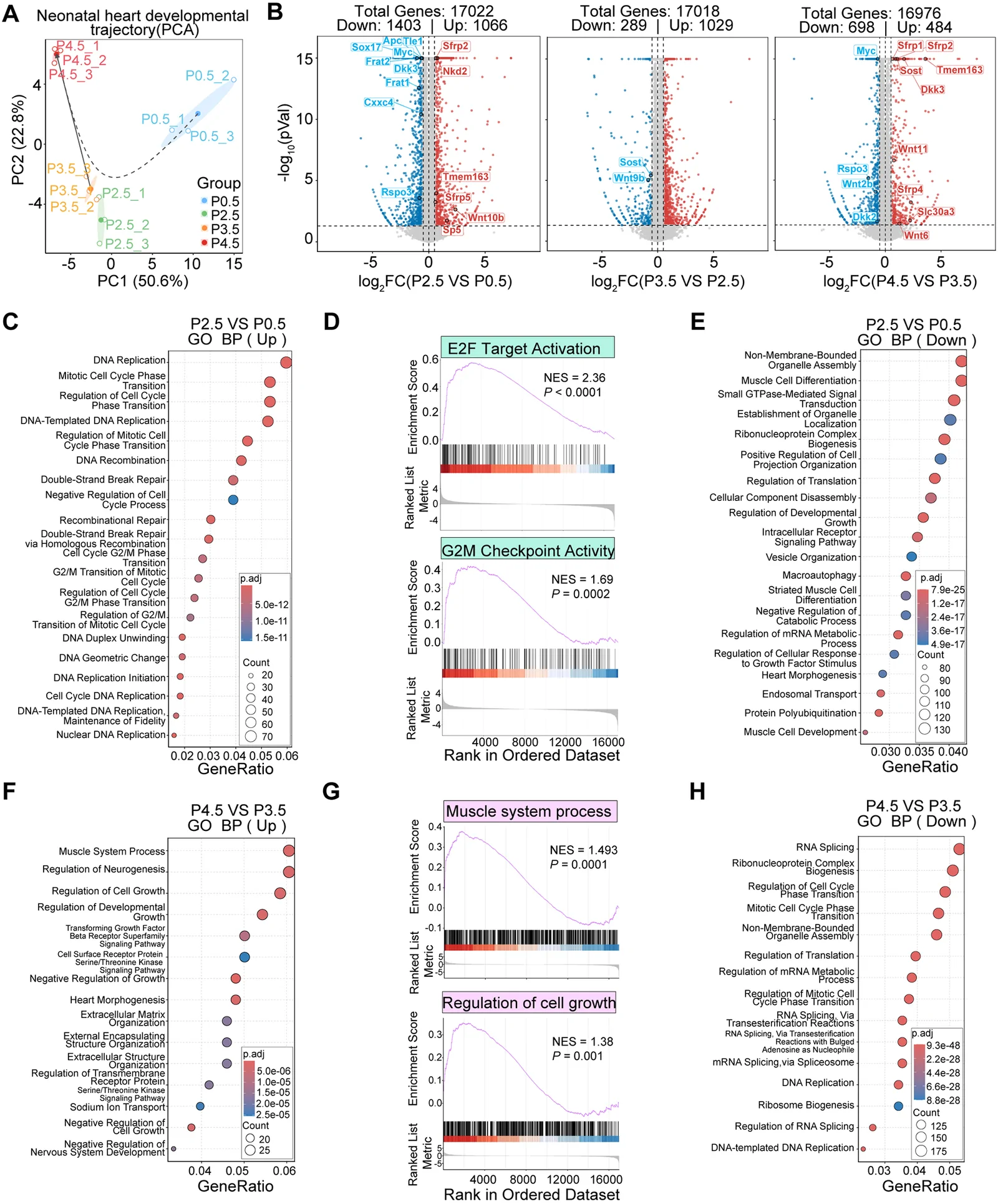
**Early postnatal mouse hearts exhibit stage-specific transcriptional programs associated with cell cycle and muscle growth.** (A) Principal component analysis (PCA) of whole-heart transcriptomes (*n*=3 biological replicates per stage). (B) Volcano plots of differential expression between consecutive stages. Red and blue dots indicate significantly upregulated and downregulated genes, respectively; gray dots indicate genes that did not meet the significance threshold. Selected development- and Wnt-associated genes are labeled. (C) Gene Ontology Biological Process (GO-BP) enrichment analysis of genes upregulated in P2.5 hearts relative to P0.5 hearts. Cell-cycle, DNA replication and chromosome segregation-related processes were enriched. (D) Gene Set Enrichment Analysis (GSEA) showing positive enrichment of Hallmark E2F Targets and Hallmark G2 M Checkpoint gene sets in P2.5 hearts compared with P0.5 hearts. NES, normalized enrichment score. (E) GO-BP enrichment analysis of genes downregulated in P2.5 hearts relative to P0.5 hearts. (F) GO-BP enrichment analysis of genes upregulated in P4.5 hearts relative to P3.5 hearts. Muscle system process, regulation of developmental growth, extracellular matrix organization and related processes were enriched at this transition. (G) GSEA showing positive enrichment of muscle system process and regulation of cell growth gene sets in P4.5 hearts compared with P3.5 hearts. (H) GO-BP enrichment analysis of genes downregulated in P4.5 hearts relative to P3.5 hearts, including RNA splicing, ribonucleoprotein complex biogenesis and cell-cycle-related processes. *P*<0.05 was considered statistically significant.

The transition from P3.5 to P4.5 was characterized by a distinct transcriptional shift. Genes upregulated at P4.5 were enriched for biological processes related to muscle system process, regulation of developmental growth, extracellular matrix organization and regulation of cell growth (Fig. 2F). GSEA further confirmed positive enrichment of muscle system process and regulation of cell growth gene sets at P4.5 compared with P3.5 (Fig. 2G). Conversely, genes downregulated at P4.5 were enriched for RNA splicing, ribonucleoprotein complex biogenesis, DNA replication and cell cycle-related processes (Fig. 2H).

Together, these morphological and transcriptomic data suggest that early postnatal heart development undergoes a stage-specific shift from cell-cycle-associated transcriptional activity toward muscle growth-associated remodeling, coinciding with cardiomyocyte enlargement and increased binucleation between P3.5 and P4.5. Given this pronounced developmental transition, we next examined whether Wnt/β-catenin- and BMP/SMAD-related signaling components displayed coordinated stage-dependent changes during early postnatal heart development.

### Stage-dependent changes in Wnt/β-catenin- and BMP/SMAD-related signaling during early postnatal heart development

Given the pronounced transcriptional and morphological transition between P3.5 and P4.5, we next examined whether Wnt/β-catenin- and BMP/SMAD-related signaling components displayed coordinated stage-dependent changes during early postnatal heart development. Heatmap analysis of whole-heart RNA-seq data revealed dynamic expression patterns of Wnt pathway-associated genes and BMP pathway-associated genes from P0.5 to P4.5 (Fig. 3A,B). Within the Wnt-associated gene set, several development-related transcriptional regulators, including *Sox1*, *Sox2*, *Zic1*, *Olig1* and *Olig2*, showed relatively higher expression around P3.5 and declined by P4.5. This pattern suggests that the P3.5-to-P4.5 transition is accompanied by remodeling of a development-associated transcriptional module, although the cellular origin and functional significance of this module require further resolution.

**Fig. 3.**
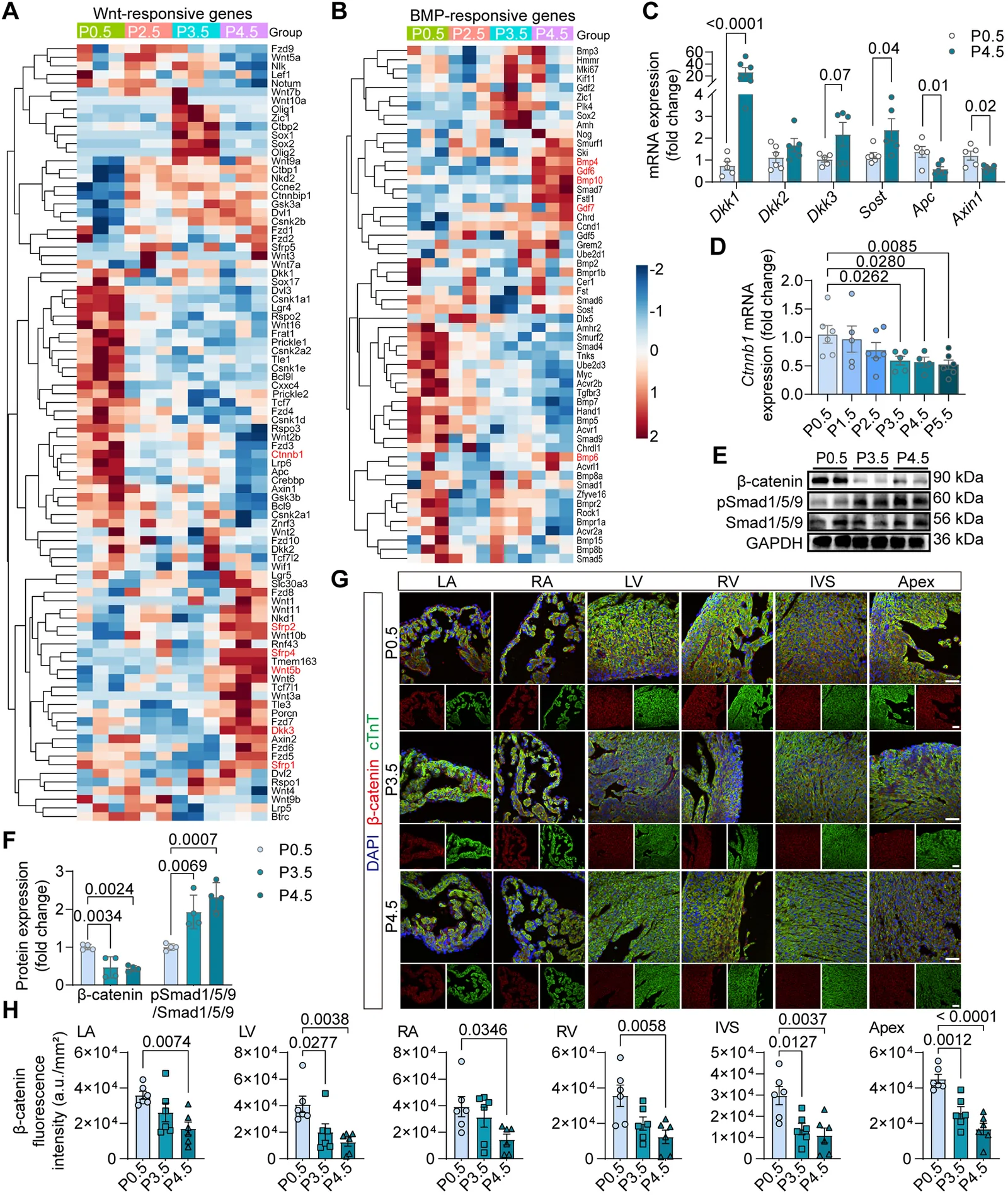
**Stage-dependent changes in Wnt/β-catenin and BMP/SMAD-related signaling during early postnatal heart development.** (A,B) Heatmaps of Wnt- (A) and BMP- (B) associated genes (*n*=3 per stage). (C) qPCR of *Dkk1*, *Dkk2*, *Dkk3*, *Sost, Apc* and *Axin1* in P0.5 and P4.5 hearts. Sample sizes are as follows: *n*=5 for *Dkk1* P0.5, *Dkk2* P4.5, *Dkk3* P0.5, *Dkk3* P4.5, *Sost* P4.5, *Apc* P0.5 and *Axin1* P0.5; *n*=6 for *Dkk1* P4.5, *Dkk2* P0.5, *Sost* P0.5, *Apc* P4.5 and *Axin1* P4.5. (D) qPCR analysis of *Ctnnb1* mRNA expression in hearts from P0.5 to P5.5 (P0.5, *n*=6; P1.5, *n*=5; P2.5, *n*=6; P3.5, *n*=5; P4.5, *n*=4; P5.5, *n*=6). (E,F) Representative western blots (E) and corresponding quantification (F) of β-catenin, phosphorylated SMAD1/5/9, total SMAD1/5/9 and GAPDH in neonatal hearts at P0.5, P3.5 and P4.5, showing β-catenin levels and the pSMAD1/5/9-to-total SMAD1/5/9 ratio (*n*=4 per stage). (G) Representative immunofluorescence images of β-catenin and cTnT (representative of *n*=6 independent experiments). Regional images are shown for the left atrium (LA), right atrium (RA), left ventricle (LV), right ventricle (RV), interventricular septum (IVS) and cardiac apex. DAPI was used to label nuclei. Scale bars: 50 μm for regional images; 5 μm for magnified images. (H) Semi-quantitative analysis of β-catenin immunofluorescence intensity in different cardiac regions at P0.5, P3.5 and P4.5, including LA, RA, LV, RV, IVS and cardiac apex (*n*=6 biological samples per stage). Data are presented as mean±s.e.m. For comparisons between two groups (C), statistical significance was assessed using an unpaired two-tailed Student's *t*-test. Multiple-stage comparisons were made using one-way ANOVA with Fisher's LSD (D), Dunnett's test against P0.5 (F), or Dunnett's test against P0.5 within each cardiac region (H). *P*<0.05 was considered statistically significant.

Given the well-established role of activated Wnt/β-catenin signaling in upregulating BMP antagonists (Im et al., 2007), which indirectly suppressing BMP pathway activity, and considering that BMP signaling serves as a key upstream suppressor of *Zic1* and *Sox2* expression (Mizuseki et al., 1998; Aruga and Mikoshiba, 2011), we hypothesized that reactivation of BMP signaling at P4.5 mediates closure of the *Sox/Zic/Olig*-dependent plasticity window. This hypothesis was supported by our observation that the module was transcriptionally upregulated at P3.5 and then synchronously downregulated at P4.5, together with a concomitant increase in components of the BMP signaling axis. BMP pathway-associated genes also showed dynamic postnatal regulation (Fig. 3B). These findings suggest that a self-buffering BMP signaling module is established during this crucial period, and that loss of Wnt/β-catenin signaling, rather than BMP activation alone, serves as the primary trigger for collapse of this plasticity window, with BMP signaling likely reinforcing the transition toward a postnatal heart development.

Consistently, qPCR analysis showed stage-dependent regulation of selected Wnt pathway modulators. *Dkk1*, *Dkk3* and *Sost* were increased at P4.5 relative to P0.5, whereas *Apc* and *Axin1* were reduced (Fig. 3C). *Ctnnb1* mRNA expression decreased progressively during early postnatal development, with significant reductions from P3.5 onwards (Fig. 3D). Western blot analysis further showed that β-catenin protein abundance was reduced at P3.5 and P4.5 compared with P0.5, whereas the phosphorylated SMAD1/5/9-to-total SMAD1/5/9 ratio was increased (Fig. 3E,F). These data suggest a developmental decrease in β-catenin abundance together with increased BMP/SMAD signaling activity. Consistently, immunofluorescence staining for β-catenin and cTnT showed β-catenin signals within cTnT-positive myocardial regions across P0.5, P3.5 and P4.5, and semi-quantitative analysis of β-catenin fluorescence intensity in these regions showed a significant reduction at P3.5 and P4.5 compared with P0.5 (Fig. 3G,H). Additional regional immunofluorescence images further demonstrated β-catenin signals across multiple cardiac regions, where they spatially overlapped with cTnT-positive myocardium (Fig. S1B). Line-scan fluorescence intensity profiles across selected myocardial regions at P0.5, P3.5 and P4.5 further illustrated the spatial relationship between β-catenin and cTnT signals (Fig. S1C). Together, these results identify a reciprocal signaling pattern during early postnatal heart development, characterized by declining β-catenin abundance and increasing SMAD1/5/9 phosphorylation as the heart transitions toward a muscle growth-associated transcriptional state.

### Disruption of β-catenin/CBP signaling enhances early postnatal cardiac growth and single-nucleated cardiomyocyte enlargement

To test whether β-catenin/CBP-associated signaling contributes to early postnatal cardiac growth, neonatal mice were treated daily with ICG-001 from P0.5 to P2.5 and analyzed at P3.5. ICG-001 treatment did not significantly alter body weight but increased heart weight, HW/BW ratio and ventricular weight, with a trend toward increased VW/BW ratio (Fig. 4A-E). These findings indicate that pharmacological disruption of β-catenin/CBP signaling during the early postnatal period enhances cardiac growth relative to body size. We next assessed whether this increase in cardiac growth was associated with changes in cardiomyocyte nuclearity, estimated cardiomyocyte number or cardiomyocyte size. ICG-001 treatment did not significantly alter the proportion of SN and DN cardiomyocytes or estimated ventricular cardiomyocyte number (Fig. 4F,G). Morphometric analysis showed that ICG-001 increased SN-CM length and estimated SN-CM volume, whereas SN-CM width was not significantly changed (Fig. 4H-J). In contrast, DN-CM length, width and estimated volume were not significantly altered by ICG-001 treatment (Fig. 4K-M). These results suggest that the increase in cardiac growth after ICG-001 treatment is associated primarily with enlargement of SN cardiomyocytes rather than increased cardiomyocyte number or accelerated binucleation.

**Fig. 4.**
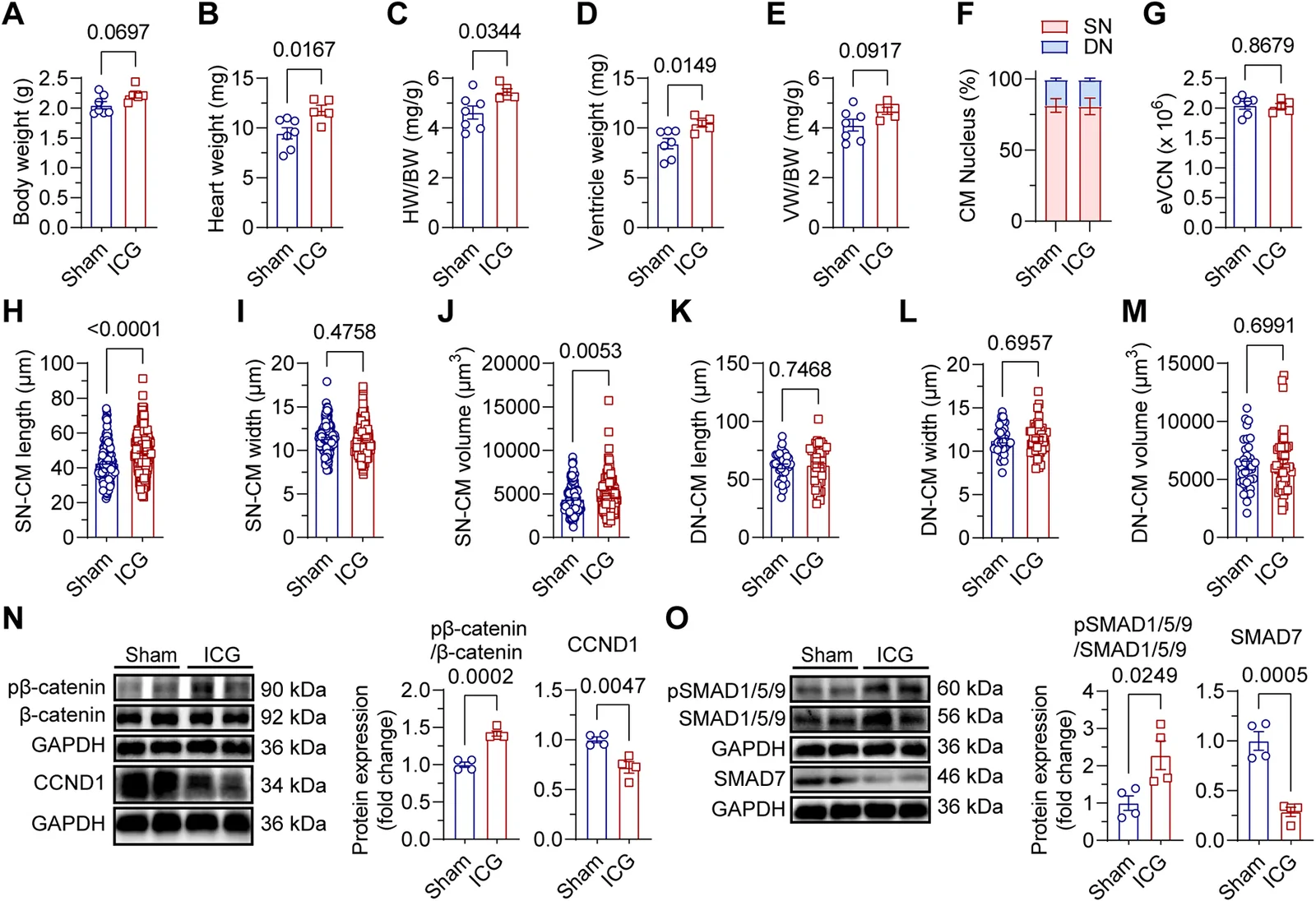
**Disruption of β-catenin/CBP signaling enhances early postnatal cardiac growth and single-nucleated cardiomyocyte enlargement.** (A) Body weight after vehicle (Sham) or ICG-001 treatment (Sham, *n*=7; ICG, *n*=5). (B-E) Assessment of cardiac growth at P3.5, including heart weight (B), heart weight-to-body weight ratio (HW/BW; C), ventricular weight (D) and ventricular weight-to-body weight ratio (VW/BW; E). (F) Quantification of single-nucleated (SN) and double-nucleated (DN) cardiomyocytes in vehicle- and ICG-001-treated hearts at P3.5. (G) Estimated ventricular cardiomyocyte number (eVCN) in vehicle- and ICG-001-treated hearts at P3.5 (Sham, *n*=6; ICG, *n*=5). (H-J) Morphometric analysis of SN cardiomyocytes, including cell length (H), cell width (I) and estimated cell volume (J). (K-M) Morphometric analysis of DN cardiomyocytes, including cell length (K), cell width (L) and estimated cell volume (M). Data are individual cell measurements from *n*=3 hearts per group, with each dot representing one cardiomyocyte. (N) Representative western blots of phospho-β-catenin (Ser33), total β-catenin, CCND1 and GAPDH, with quantification of the phospho-β-catenin-to-total β-catenin ratio and CCND1 protein levels, in vehicle- and ICG-001-treated hearts. (O) Representative western blots of phospho-SMAD1 (Ser463/465)/SMAD5 (Ser463/465)/SMAD9 (Ser465/467), total SMAD1/5/9, SMAD7 and GAPDH, with quantification of the p-SMAD1/5/9-to-total SMAD1/5/9 ratio and SMAD7 protein levels, in vehicle- and ICG-001-treated hearts (*n*=4 per group). Data are presented as mean±s.e.m. Unpaired two-tailed Student's *t*-tests were used for statistical comparisons. *P*<0.05 was considered statistically significant.

At the signaling level, ICG-001 increased β-catenin phosphorylation at Ser33, a degradation-associated site, relative to total β-catenin, consistent with reduced β-catenin stability. ICG-001 also reduced CCND1 protein levels, supporting attenuation of β-catenin-associated cell-cycle output. In parallel, ICG-001 increased phosphorylated SMAD1/5/9 relative to total SMAD1/5/9 and reduced SMAD7 protein levels (Fig. 4N,O). We further examined whether ICG-001 treatment reproduced selected transcriptional features of the developmental P3.5-to-P4.5 transition. A P3.5-enriched Wnt-responsive and development-associated gene subset, including *Sox1*, *Sox2*, *Zic1*, *Olig1* and *Olig2*, declined at P4.5 and was similarly reduced after ICG-001 treatment (Fig. S2A). Notably, Ctbp2, a reported negative regulator of β-catenin-dependent transcription (Kim et al., 2017), remained relatively high after ICG-001 treatment (Fig. S2A), a pattern that may contribute to the attenuated β-catenin-associated transcriptional state. In addition, ICG-001 treatment was associated with broad remodeling of BMP-responsive genes (Fig. S2B) and partial overlap with the developmental P3.5-to-P4.5 transition in cardiac growth- and remodeling-associated genes (Fig. S2D).

Together, these findings indicate that β-catenin/CBP disruption enhances early postnatal cardiac growth and SN-CM enlargement, accompanied by increased degradation-associated β-catenin phosphorylation, reduced CCND1 expression, enhanced SMAD1/5/9 phosphorylation and partial transcriptional convergence with the developmental P3.5-to-P4.5 transition.

### *Ctnnb1* knockdown enhances early postnatal cardiomyocyte enlargement and reduces estimated ventricular cardiomyocyte number

To further test whether reduced β-catenin abundance affects early postnatal cardiac growth, neonatal mice were treated with *Ctnnb1* siRNA and analyzed during the early postnatal period. *Ctnnb1* siRNA significantly reduced *Ctnnb1* mRNA expression and β-catenin protein abundance compared with negative control siRNA (Fig. 5A-C). Body weight was not significantly altered, whereas heart weight and ventricular weight were increased after *Ctnnb1* knockdown, with no significant change in HW/BW or VW/BW ratios (Fig. 5D-H). We next examined cardiomyocyte nuclearity, size and estimated ventricular cardiomyocyte number. *Ctnnb1* knockdown did not markedly alter the proportion of SN and DN cardiomyocytes (Fig. 5I). However, morphometric analysis showed significant increases in SN-CM length and estimated volume, with a trend toward increased SN-CM width (Fig. 5J-L). DN-CMs also showed increased length, width and estimated volume after *Ctnnb1* knockdown (Fig. 5M–O). In contrast to the increase in cardiomyocyte size, eVCN was significantly reduced in Ctnnb1 siRNA-treated hearts (Fig. 5P). These results indicate that systemic *Ctnnb1* knockdown is associated with enhanced cardiomyocyte enlargement but reduced estimated ventricular cardiomyocyte number.

**Fig. 5.**
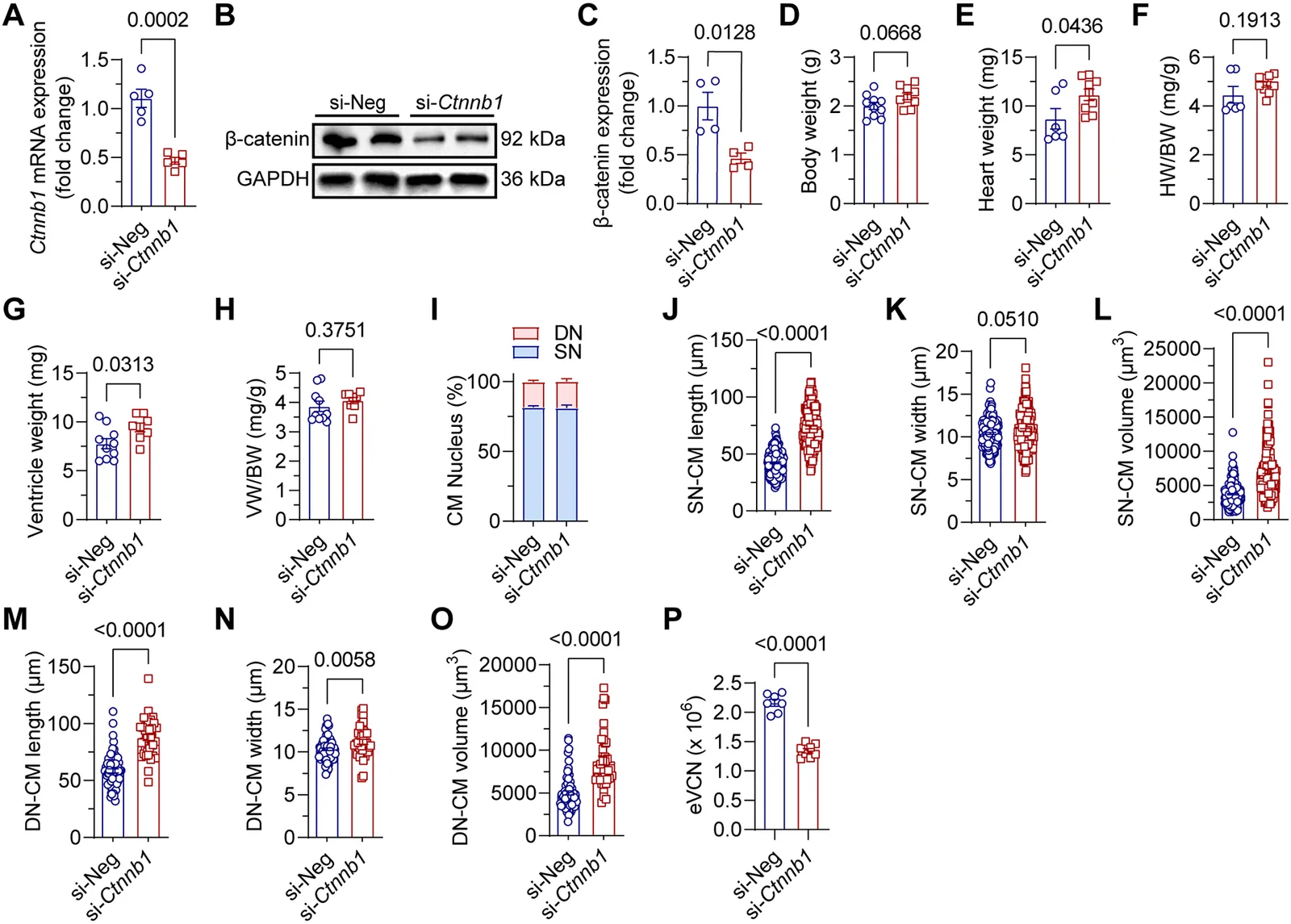
***Ctnnb1* knockdown enhances early postnatal cardiac growth and cardiomyocyte enlargement while reducing estimated ventricular cardiomyocyte number.** (A) qPCR analysis of *Ctnnb1* mRNA expression (*n*=5 per group). (B) Representative western blots showing β-catenin and GAPDH protein levels in negative control siRNA- and *Ctnnb1* siRNA-treated hearts. (C) Quantification of β-catenin protein levels shown in B (*n*=4 per group). (D) Body weight of neonatal mice after treatment with negative control siRNA or *Ctnnb1* siRNA (negative control, *n*=10; *Ctnnb1* siRNA, *n*=9). (E-H) Assessment of cardiac growth after *Ctnnb1* knockdown. Exact sample sizes are as follows. Heart weight (E) and HW/BW (F): negative control, *n*=6; *Ctnnb1* siRNA, *n*=9. Ventricular weight (G) and VW/BW (H): negative control, *n*=10; *Ctnnb1* siRNA, *n*=8. (I) Quantification of single-nucleated (SN) and double-nucleated (DN) cardiomyocytes in negative control siRNA- and *Ctnnb1* siRNA-treated hearts. (J-O) Morphometric analysis of SN and DN cardiomyocytes (J-L, SN; M-O, DN). Data are individual cell measurements from *n*=3 hearts per group, with each dot representing one cardiomyocyte. (P) Estimated ventricular cardiomyocyte number (eVCN) (negative control, *n*=7; *Ctnnb1* siRNA, *n*=8). Data are presented as mean±s.e.m. Unpaired two-tailed Student's *t*-tests were used for statistical comparisons. *P*<0.05 was considered statistically significant.

At the transcriptional level, *Ctnnb1* knockdown also reduced the P3.5-enriched Wnt-responsive and development-associated gene subset that declined during the developmental P3.5-to-P4.5 transition (Fig. S2A). In parallel, *Ctnnb1* knockdown was associated with remodeling of BMP-responsive genes, including changes in BMP ligands, receptors, SMAD-related mediators and intracellular feedback regulators (Fig. S2C). Analysis of representative cardiac growth- and remodeling-associated genes further showed partial overlap between *Ctnnb1* knockdown and the developmental P3.5-to-P4.5 transition (Fig. S2D).

Together, these findings indicate that reducing β-catenin abundance enhances cardiomyocyte enlargement during early postnatal heart development while decreasing estimated ventricular cardiomyocyte number. In combination with the ICG-001 results, these data support a model in which reduced β-catenin-associated signaling favors a shift from cell-cycle-associated transcriptional activity toward growth-associated cardiac remodeling during the early postnatal transition.

### Pharmacological stabilization of β-catenin increases cardiomyocyte cell-cycle activity while limiting cardiomyocyte enlargement

To determine whether activation of β-catenin-associated signaling produces effects opposite to β-catenin attenuation, neonatal mice were treated with CHIR-99021 (CHIR), a GSK-3 inhibitor, from P0.5 to P2.5 and analyzed at P3.5. Western blot analysis further showed reduced β-catenin phosphorylation at Ser33, a degradation-associated site, consistent with increased β-catenin stabilization (Fig. 6A). CHIR treatment did not significantly alter body weight or absolute ventricular weight, but significantly increased the VW/BW ratio (Fig. 6B-D), suggesting enhanced ventricular growth relative to body size. Morphometric analysis revealed that CHIR treatment increased cell length but reduced cell width in both SN-CMs and DN-CMs. These dimensional changes resulted in a significant reduction in estimated cell volume in both cardiomyocyte populations (Fig. 6E-J), consistent with recent observations that proliferating cardiomyocytes undergo distinct morphological remodeling (Huang et al., 2024). In contrast to the reduction in cardiomyocyte volume, eVCN was significantly increased after CHIR treatment (Fig. 6K), suggesting that β-catenin stabilization shifts early postnatal cardiac growth toward increased cardiomyocyte number rather than cardiomyocyte enlargement.

**Fig. 6.**
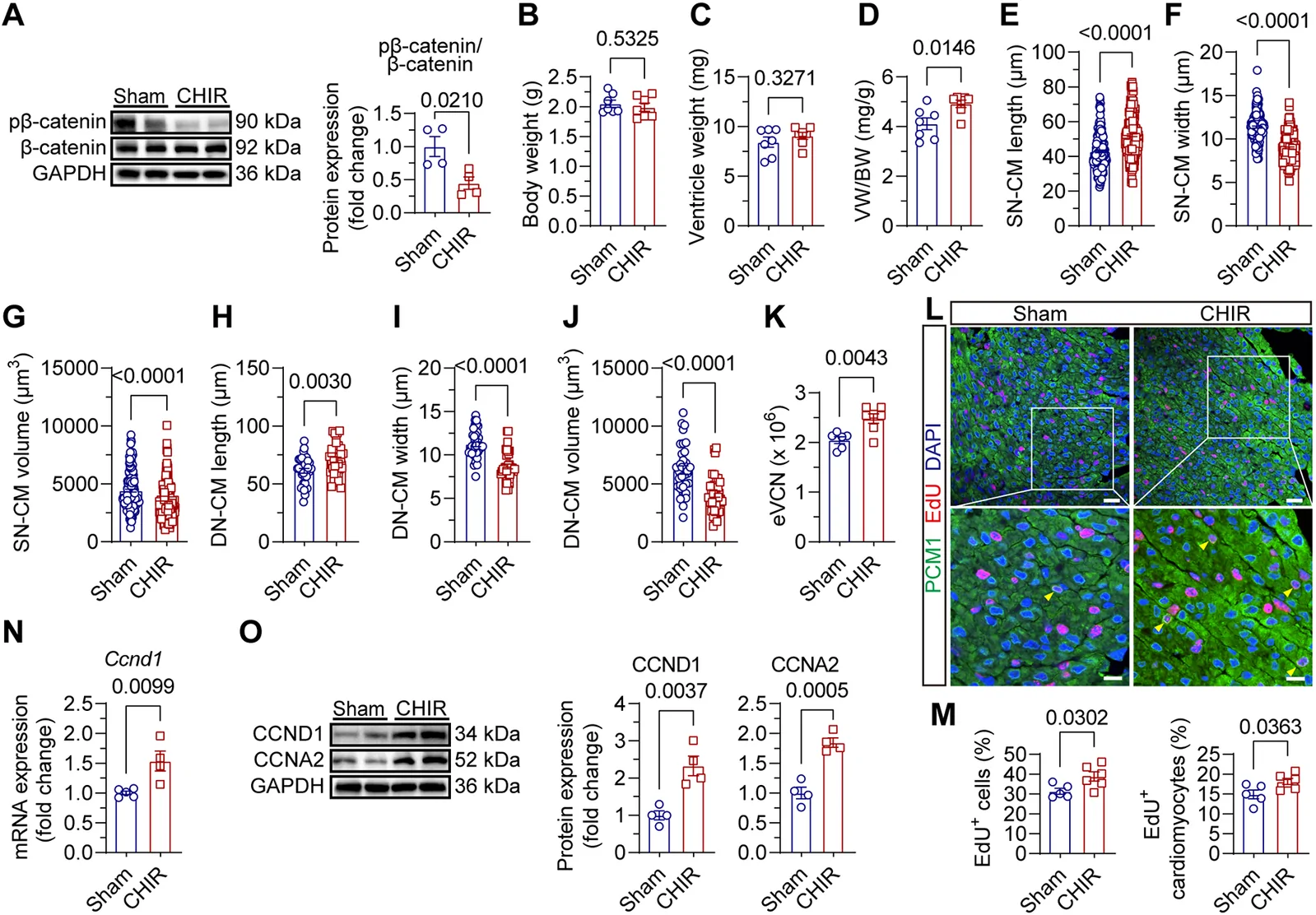
**CHIR-99021 enhances cardiomyocyte proliferation-associated features while limiting cardiomyocyte enlargement in the postnatal heart.** (A) Representative western blots of phospho-β-catenin (Ser33), total β-catenin and GAPDH, with quantification of the phospho-β-catenin-to-total β-catenin ratio, in vehicle- and CHIR-99021 (CHIR)-treated hearts (*n*=4 per group). (B) Body weight of neonatal mice measured at P3.5 after daily treatment with vehicle or CHIR from P0.5 to P2.5 (*n*=7 per group). (C,D) Assessment of ventricular growth after CHIR treatment, including ventricular weight (C) and ventricular weight-to-body weight ratio (VW/BW; D) (*n*=7 per group). (E-J) Morphometric analysis of single-nucleated (SN) and double-nucleated (DN) cardiomyocytes (E-G, SN; H-J, DN). Data are individual cell measurements from *n*=3 hearts per group (each dot represents data from one cardiomyocyte). (K) Estimated ventricular cardiomyocyte number (eVCN) in Sham and CHIR group (Sham, *n*=6; CHIR, *n*=7). (L) Representative immunofluorescence images showing EdU incorporation and PCM1 staining in heart sections from vehicle- and CHIR-treated mice. Yellow arrowheads indicate EdU-positive, PCM1-positive cardiomyocytes. Nuclei were counterstained with DAPI. Scale bars: 20 μm; 10 μm in enlarged images. (M) Percentage of EdU-positive cells and EdU-positive cardiomyocytes (Sham, *n*=5; CHIR, *n*=6). (N) qPCR analysis of *Ccnd1* mRNA expression in vehicle- and CHIR-treated hearts (*n*=4 per group). (O) Representative western blots and corresponding quantification of CCND1 and CCNA2 protein levels in vehicle- and CHIR-treated hearts (*n*=4 per group). Data are presented as mean±s.e.m. Unpaired two-tailed Student's *t*-tests were used for statistical comparisons. *P*<0.05 was considered statistically significant.

EdU incorporation was also detected in PCM1-positive cardiomyocytes. Both the overall proportion of proliferating cells and the percentage of EdU-positive cardiomyocytes were increased (Fig. 6L,M). At the molecular level, CHIR increased *Ccnd1* mRNA expression (Fig. 6N). Western blot analysis further showed increased CCND1 and CCNA2 protein levels (Fig. 6O).

Together, these findings indicate that pharmacological stabilization of β-catenin during the early postnatal period enhances cardiomyocyte cell-cycle-associated activity and increases estimated ventricular cardiomyocyte number, while limiting cardiomyocyte enlargement. These results complement the β-catenin attenuation experiments and support a model in which β-catenin-associated signaling influences the balance between cardiomyocyte number expansion and cardiomyocyte growth during early postnatal heart development.

### Single-cell transcriptomic landscape reveals multi-lineage coordination of Wnt/BMP signaling

To resolve the cellular context of the developmental programs identified in whole-heart transcriptomes, we integrated public mouse cardiac single-cell RNA-seq datasets spanning E8.5 to P6. After quality control, doublet removal, normalization and batch correction, 100,159 cells were retained for analysis. UMAP visualization showed integration across datasets, stages and samples, and major cardiac cell populations were annotated based on canonical marker genes (Fig. 7A and Fig. S3). Marker analysis showed that the Sox, Zic and Olig-containing developmental gene subset identified in bulk RNA-seq was enriched predominantly in an early neural and glial progenitor-like population, with limited expression in cardiomyocytes and other major cardiac cell types (Fig. 7B).

**Fig. 7.**
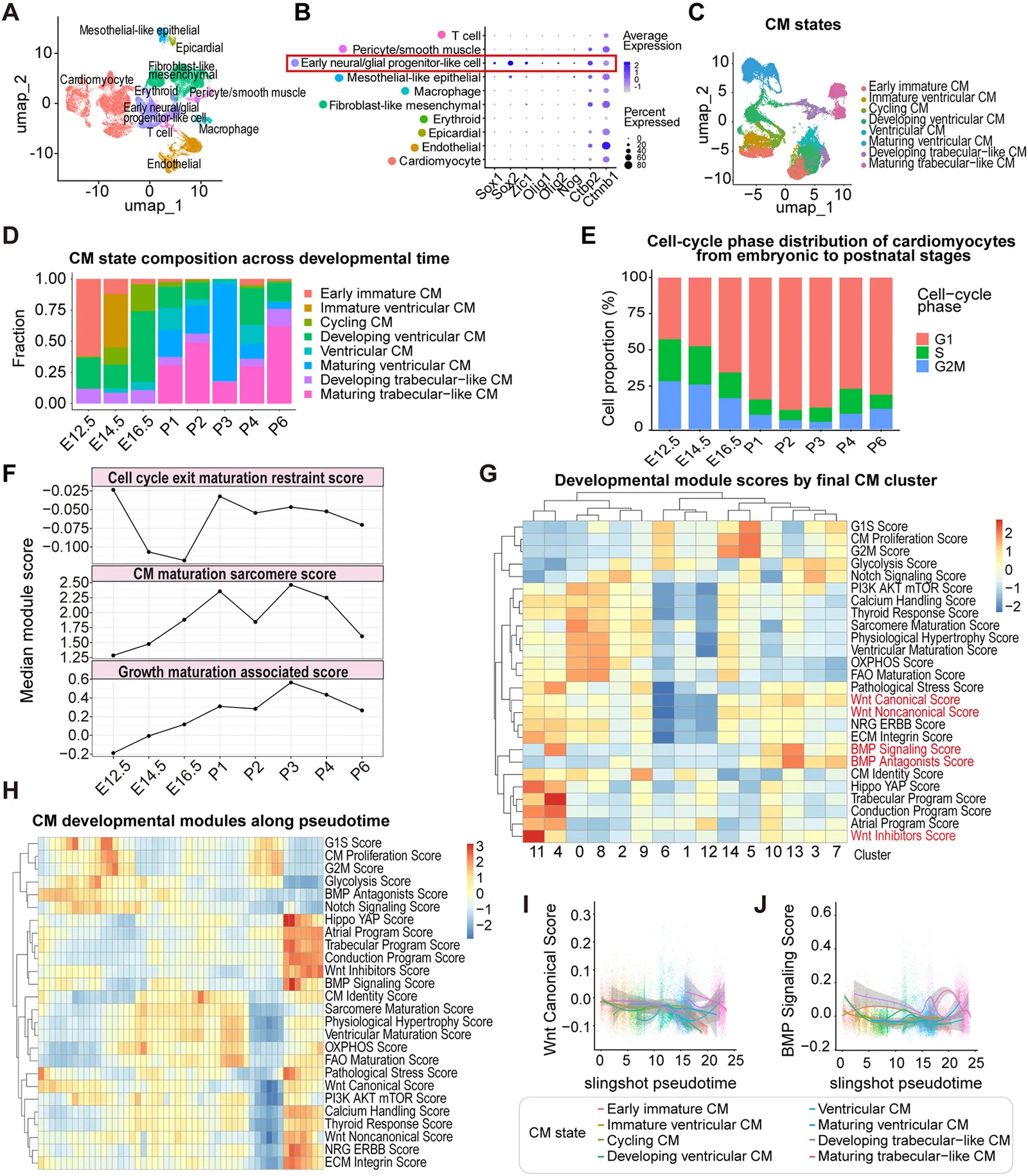
**Single-cell transcriptomics resolves the cellular origin of developmental gene modules and defines cardiomyocyte developmental trajectories.** (A) UMAP visualization of the integrated public mouse cardiac single-cell RNA-seq dataset, colored by major annotated cell types. Major populations included cardiomyocytes, endothelial cells, fibroblast-like mesenchymal cells, epicardial cells, pericyte and smooth muscle cells, macrophages, T cells, early neural and glial progenitor-like cells, mesothelial-like epithelial cells, and erythroid cells. (B) Dot plot showing the expression of selected marker genes across major annotated cell types. The early neural and glial progenitor-like population showed enriched expression of *Sox1*, *Sox2*, *Zic1*, *Olig1* and *Olig2*. (C) UMAP visualization of reclustered cardiomyocytes, colored by annotated cardiomyocyte developmental states, including early immature cardiomyocytes, immature ventricular cardiomyocytes, cycling cardiomyocytes, developing ventricular cardiomyocytes, ventricular cardiomyocytes, maturing ventricular cardiomyocytes, developing trabecular-like cardiomyocytes and maturing trabecular-like cardiomyocytes. (D) Relative composition of cardiomyocyte developmental states across embryonic and postnatal stages from E12.5 to P6. (E) Distribution of cardiomyocytes assigned to G1, S and G2 M phases across developmental stages from E12.5 to P6. (F) Stage-wise changes in selected cardiomyocyte developmental module scores, including cell-cycle exit and maturation-restraint score, sarcomere maturation score, and growth-associated maturation score. (G) Heatmap showing developmental and signaling module scores across cardiomyocyte subclusters. The corresponding cluster annotations are provided in Fig. S4. (H) Heatmap showing cardiomyocyte developmental module scores ordered along Slingshot pseudotime. (I,J) Smoothed trends of canonical Wnt signaling score (I) and BMP signaling score (J) along Slingshot pseudotime, stratified by cardiomyocyte developmental state.

Cardiomyocytes were then extracted and reclustered for developmental state analysis from E12.5 to P6, yielding 33,136 high-confidence cardiomyocytes after quality filtering (Fig. S4). Cardiomyocyte subclusters were annotated into distinct developmental states based on marker gene expression (Fig. 7C and Fig. S4E). This analysis provided a single-cell framework to interpret the P3.5-to-P4.5 transition identified in our morphometric and bulk transcriptomic analyses. Around this interval, P3 hearts were enriched for maturing ventricular cardiomyocytes, whereas P4 hearts showed a relative reduction in this state, together with increased representation of developing ventricular cardiomyocytes, and detectable early immature and cycling cardiomyocyte states (Fig. 7D). Cell-cycle phase analysis showed an overall developmental decline in S- and G2M-phase cardiomyocytes from embryonic to postnatal stages, but a modest increase in S and G2M assignment around P4 compared with P3 (Fig. 7E). In parallel, cell type-resolved analysis showed reduced cardiomyocyte expression of *Ctnnb1* around P4, suggesting attenuation of β-catenin-associated signaling within cardiomyocytes during this transition (Fig. S5). These changes occurred near the developmental window in which binucleated cardiomyocytes increased and cardiomyocyte enlargement became prominent in our experimental dataset (Fig. 1G-O). Together, these observations suggest that the P3.5-to-P4.5 interval involves a heterogeneous cardiomyocyte transition, in which residual cell cycle-associated states co-exist with emerging growth-associated remodeling under a declining β-catenin-associated signaling context.

Module score analysis further supported the developmental transition identified in the morphometric and bulk transcriptomic analyses. Across developmental stages, cardiomyocyte cell cycle-associated and maturation-restraint scores decreased, whereas sarcomere maturation and growth-associated maturation scores increased during the embryonic-to-postnatal transition (Fig. 7F). Heatmap analysis across cardiomyocyte subclusters revealed distinct developmental and signaling module-score patterns, with corresponding annotations provided in Fig. S4. Interestingly, the cardiomyocyte clusters with high scores for both canonical Wnt and BMP signaling were largely overlapping and enriched in the same subpopulations: cluster 7 (early immature CMs), cluster 13 (developing ventricular CMs) and cluster 10 (developing trabecular-like CMs) (Fig. 7G). This co-enrichment suggests that canonical Wnt and BMP signaling are not mutually exclusive but rather co-activated in specific immature and developing cardiomyocyte states, potentially to coordinate proliferation with early maturation cues. Notably, clusters 4 and 11, annotated as maturing trabecular-like cardiomyocytes, exhibited relatively high scores for canonical Wnt, non-canonical Wnt, Wnt inhibitor and BMP signaling, indicating that these programs remain selectively enriched in specific trabecular-like cardiomyocyte states during developmental progression. Proliferation-associated subclusters were enriched for cell-cycle programs, whereas more differentiated cardiomyocyte states showed higher structural, metabolic and growth-associated scores.

Pseudotime analysis further organized cardiomyocyte states along a continuous developmental trajectory. Proliferation-associated modules, including G1/S, G2/M and cardiomyocyte proliferation scores, were enriched at early pseudotime and declined progressively, whereas sarcomere maturation, calcium handling, metabolic maturation, physiological hypertrophy and ventricular maturation scores increased toward intermediate-to-late pseudotime regions (Fig. 7H). This pattern is consistent with the transition from cell cycle-associated transcriptional activity toward structural and growth-associated cardiomyocyte remodeling observed in our bulk RNA-seq and morphometric analyses.

Signaling modules also displayed ordered pseudotime dynamics. Canonical Wnt signaling scores remained relatively low overall but fluctuated across cardiomyocyte states, whereas Wnt inhibitor scores increased prominently at late pseudotime (Fig. 7H,I). BMP signaling scores also rose toward late pseudotime and were enriched in more developmentally advanced cardiomyocyte states (Fig. 7H,J). These findings suggest that cardiomyocyte developmental progression is accompanied by declining proliferative programs, increasing structural and growth-associated programs, and a late shift toward BMP-related and Wnt-inhibitory signaling features.

## DISCUSSION

In this study, we define a multi-lineage signaling coordination that coordinates the early postnatal transition from cardiomyocyte proliferation-associated growth to cell hypertrophic enlargement. Our morphometric and transcriptomic data identify the P3.5-to-P4.5 interval as a crucial window in which cardiomyocyte binucleation and volume expansion coincide with a whole-heart transcriptional shift from cell-cycle programs toward muscle growth-associated remodeling. Within this window, β-catenin abundance decreases while SMAD1/5/9 phosphorylation increases, suggesting a reciprocal relationship between Wnt/β-catenin and BMP/SMAD pathways. Functional perturbations support this model: attenuating β-catenin signaling (ICG-001 or siRNA) promotes cardiomyocyte enlargement and reduces estimated ventricular cardiomyocyte number, whereas β-catenin stabilization (CHIR-99021) enhances cell-cycle activity and increases estimated cardiomyocyte number while limiting enlargement. Thus, β-catenin-associated signaling acts as a rheostat that balances the mode of early postnatal cardiac growth.

### A multi-cell type interplay between Wnt and BMP signaling underlies the growth transition

Single-cell transcriptomic integration unexpectedly revealed that the Wnt-related developmental gene module (*Sox2*, *Zic1* and *Olig2*) is not expressed in cardiomyocytes but is enriched in early neural/glial progenitor cells (Fig. 7B). However, the broader Wnt-to-BMP transition engages multiple cardiac cell types in a temporally coordinated manner. At P2, non-canonical Wnt ligands (Wnt5a and Wnt11) are expressed in epicardial, pericyte/smooth muscle, endothelial and fibroblast-like mesenchymal cells, coinciding with the peak of cardiomyocyte proliferation. Notably, both ligands are also expressed in embryonic cardiomyocytes (E12.5-E14.5), suggesting a developmental shift from a cardiomyocyte-intrinsic to a non-myocyte-derived source of non-canonical Wnt signaling as the heart matures. By P6, BMP transduction components (Smad1/4/5/7) and TAK1-interacting genes (Tab1-3) reach their highest activity in cardiomyocytes, while also being upregulated in endothelial and neural/glial cells. This sequential activation, with a Wnt-dominant phase in non-myocytes at P2-P3 followed by a BMP-dominant phase in cardiomyocytes at P6, defines a handover rather than a simple on-off switch.

### Paracrine BMP4 from non-myocytes drives cardiomyocyte enlargement

A key mechanistic insight is the discovery of a paracrine BMP4 signaling axis. Although bulk RNA-seq detected upregulation of both Bmp4 and Bmp10 at P4.5 (Fig. 3B), single-cell resolution revealed that Bmp10, which restricts postnatal cardiomyocyte enlargement (Chen et al., 2006a, 2004), is derived from cardiomyocytes, whereas Bmp4, a known promoter of cardiomyocyte enlargement (Sun et al., 2013; Sheng et al., 2025; Wang et al., 2015), is expressed in endothelial and neural/glial cells. In contrast, cardiomyocytes robustly upregulate BMP receptors (Bmpr1a and Bmpr2), downstream effectors (Smad4, Smad5 and Smad7), and TAK1-interacting genes. This expression pattern strongly supports a model in which BMP4 from adjacent non-myocytes activates BMP/SMAD signaling in cardiomyocytes to drive hypertrophic growth. Such paracrine crosstalk may explain why mononucleated cardiomyocytes can undergo enlargement without immediate binucleation, as cytokinesis failure is governed by separate regulatory modules (e.g. Cyclin G1 and E2f/Rb) (Porrello et al., 2011; Naqvi et al., 2014; Yan et al., 2021).

### Closure of the neural plasticity window by BMP signaling

The neural Sox/Zic/Olig-containing module is activated in a β-catenin-dependent manner around P3.5 and declines by P4.5-P6. Both ICG-001 and β-catenin siRNA prevent its normal activation, indicating that downregulation of Wnt signaling initiates the dissolution of this module. The subsequent rise in BMP signaling provides a direct mechanism to suppress Sox2 and Zic1 expression (Mizuseki et al., 1998; Aruga and Mikoshiba, 2011), thereby contributing to closure of the neural plasticity window. Of note, the transient upregulation of this low-abundance neural module at P3.5 was not captured by our single-cell RNA-seq analysis, likely due to technical characteristics of the assay, such as filtering and normalization steps, that can attenuate signals from rare or low-expression transcripts in heterogeneous cardiac tissues. Nevertheless, our single-cell data show that by P6, the same neural progenitor-like cells exhibit marked upregulation of BMP transduction components, coinciding with the loss of Sox2, Zic1 and Olig2. Together, these findings support a temporal association between declining Wnt-related activity, increasing BMP signaling and attenuation of this module (Nakashima et al., 2005; Im et al., 2007).

### Co-activator usage and context-dependent Wnt functions

Selective inhibition of β-catenin/CBP with ICG-001 induced hypertrophy mainly in mononucleated cardiomyocytes without affecting binucleation, whereas global β-catenin knockdown promoted enlargement of both mono- and binucleated cells, and reduced estimated cell number. This phenotypic divergence suggests that CBP-dependent transcription primarily licenses hypertrophic growth, while CBP-independent β-catenin functions (potentially via p300) help sustain proliferative capacity (Mazzotta et al., 2016; Foulquier et al., 2018). These findings reconcile the long-standing paradox that Wnt/β-catenin signaling can be both pro-proliferative and pro-hypertrophic depending on the cellular context and co-activator usage (Tzahor, 2007). Therapeutically, transiently reactivating the proliferative Wnt program in non-myocytes, or modulating paracrine BMP4, may offer refined strategies for cardiac repair.

### Limitations and future directions

Several limitations should be acknowledged. First, the exact paracrine factors mediating cross-talk between neural, endothelial, mesenchymal and cardiomyocyte compartments remain incompletely defined. Second, establishing a causal role for BMP signaling in cardiomyocyte enlargement would require direct gain- or loss-of-function experiments (e.g. BMP activation or inhibition), which were not performed here. Third, formal genetic proof that the Sox/Zic/Olig-containing gene subset is necessary or sufficient will require cell-type-specific perturbations (e.g. forced co-expression of Sox2, Zic1 and Olig2), and our single-cell data indicate that this module is enriched in early neural/glial cells rather than cardiomyocytes, so its functional relevance to cardiomyocyte growth remains to be determined. Fourth, we did not directly examine the effects of sustained Wnt activation (CHIR-99021) on BMP signaling or on the neural module. Fifth, we did not perform *ex vivo* manipulations on purified primary neonatal cardiomyocytes; however, existing *in vitro* literature supports a direct pro-hypertrophic effect of BMP4 on cardiomyocytes, consistent with our paracrine model. Finally, the full set of CBP/β-catenin-dependent target genes that drive growth-associated remodeling remains to be identified. Addressing these limitations will further strengthen the multi-lineage coordination framework.

### Conclusions

In summary, we have uncovered a multi-lineage interplay between Wnt and BMP signaling that coordinates early postnatal heart development. A transient Wnt-dominant phase in neural, endothelial and mesenchymal cells supports cardiomyocyte proliferation, whereas a subsequent BMP-dominant phase is driven by paracrine factor BMP4 from non-myocytes, thereby promoting cardiomyocyte enlargement and closing the neural plasticity window. This integrated, multi-cell-type signaling interplay provides a new conceptual framework for understanding how the heart switches from cardiomyocyte hyperplastic to enlargement growth and offers potential targets for regenerative medicine.

## MATERIALS AND METHODS

### Animals and neonatal treatments

All animal experiments were conducted in accordance with the guidelines of the Wenzhou Medical University Animal Care and Use Committee (wyy15854234987). C57BL/6J mice (*Mus musculus*) were purchased from the Chinese Academy of Medical Sciences (Shanghai, China). Animals were housed under controlled conditions at 22±1°C with a 12 h light/dark cycle and free access to food and water. The day of birth was designated as postnatal day 0.5 (P0.5), and *in vivo* neonatal treatments were initiated 12 h after birth. To minimize inter-litter and developmental variation, only pups from litters of 6 or 7, with body weights between 1.3 and 1.5 g and confirmed birth times within 12 h were included. Both male and female pups were used for all experiments, unless otherwise stated.

ICG-001, CHIR-99021, β-catenin siRNA or the corresponding control treatment was administered to neonatal pups by subcutaneous injection once daily for 3 consecutive days starting at P0.5. ICG-001 was purchased from MedChemExpress (MCE; HY-14428) and administered at 1.2 mg/kg/day. CHIR-99021 was purchased from MedChemExpress (MCE; HY-10182B) and administered at 10 mg/kg/day. Control pups received the corresponding vehicle solution or sterile physiological saline. β-Catenin siRNA targeting Ctnnb1 and non-targeting negative control siRNA were purchased from Guangzhou RiboBio. The catalog number for the non-targeting control siRNA was siN0000005-4-10; the catalog number for the *Ctnnb1*-targeting siRNA was siBDMV002. Lyophilized siRNA was dissolved in RNase-free water to prepare a 20 μM working solution. For *in vivo* knockdown, neonatal pups received β-catenin siRNA or control siRNA at 0.5 nmol per pup in a total volume of 25 μl by subcutaneous injection once daily for 3 consecutive days.

### Heart harvest and tissue weight measurement

Neonatal mice were euthanized by decapitation at the indicated postnatal stages. Hearts were rapidly excised and placed in ice-cold phosphate-buffered saline (PBS). After removal of surrounding non-cardiac tissues, the whole heart was gently blotted dry and weighed immediately using a precision microbalance. The atria and great vessels were then carefully removed, and the remaining ventricular tissue was weighed separately. Ventricular tissue comprised both left and right ventricular free walls and the interventricular septum. Depending on the experiment, whole-heart tissues or ventricular tissues were collected for RNA extraction, protein extraction, histological analysis or cardiomyocyte isolation. For RNA-seq analysis, whole-heart tissues were used. For ventricular weight measurement, protein extraction, qRT-PCR and cardiomyocyte isolation, ventricular tissues were used unless otherwise stated. The tissue source used for each analysis is specified in the corresponding Materials and Methods section and figure legend.

### Langendorff retrograde perfusion and isolated cardiomyocyte preparation for morphometric analysis

Neonatal mice were euthanized following intraperitoneal injection of heparin (10 μl of 1000 IU/ml) 20-30 min prior to sacrifice, as described previously (Naqvi et al., 2014; Nicks et al., 2022). The chest area was sterilized with 70% ethanol, and the anterior chest wall was carefully removed to expose the thoracic cavity. A longer segment of the aorta, approximately 0.5-1 cm, was retained to facilitate identification and cannulation. The heart was gently excised and cannulated *in situ* via the aorta using a 30G blunt needle, which was secured with a silk suture. Care was taken to avoid advancing the cannula beyond the aortic valve or into the left ventricle. The heart was then transferred to the Langendorff perfusion apparatus for enzymatic digestion. A calcium-free modified Krebs-Ringer solution was first used to flush residual blood from the coronary circulation, followed by perfusion with digestion buffer containing 0.1% collagenase II. After enzymatic digestion, the atria were removed, and the ventricles were transferred into a Petri dish containing ice-cold transfer buffer. Ventricular tissues were gently minced with forceps and mechanically dissociated by gentle trituration using a plastic transfer pipette for no more than 2 min. The resulting cell suspension was passed through a 70 μm cell strainer to remove undigested tissue fragments. The isolated cardiomyocyte-containing suspension was fixed in 2% paraformaldehyde to preserve cellular morphology. Fixed cells were collected by gentle centrifugation and deposited onto glass slides by cytocentrifugation. The cells were then stained with FITC-conjugated phalloidin to visualize the actin cytoskeleton and cell boundaries, and nuclei were counterstained with DAPI. These isolated cardiomyocyte preparations were used for morphometric analyses, including measurement of cardiomyocyte length, width, estimated volume and nucleation status.

### Cardiomyocyte size, nucleation and volume quantification

Isolated ventricular cardiomyocytes were fixed in 2% paraformaldehyde and stained with FITC-conjugated phalloidin to visualize cell boundaries. Nuclei were counterstained with DAPI. Images were acquired using identical imaging settings within each experiment. Only intact cardiomyocytes with clearly defined borders, preserved morphology and non-overlapping cell boundaries were included. Damaged, fragmented or overlapping cells were excluded. Cardiomyocyte volume was determined through microscopy and image-based morphometric analysis (Naqvi et al., 2014). Cardiomyocytes were classified as mononucleated or binucleated according to the number of DAPI-positive nuclei enclosed within a clearly defined cardiomyocyte boundary. Cell length (L) and width (W) were measured using ImageJ software. Cardiomyocyte volume (V) was estimated by approximating each cell as a cylinder using the formula: V=π×(W/2)^2^×L, where *V* represents the volume in cubic micrometers (μm^3^), *L* is cell length and *W* is cell width. For each heart, at least 100 cardiomyocytes were analyzed. The mean value from each heart was used as one biological replicate for statistical analysis. To account for nuclearity-associated differences in cell size, a weighted average cardiomyocyte volume was calculated for each heart using the following formula:

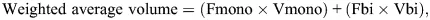
where Fmono and Fbi represent the fractions of mononucleated and binucleated cardiomyocytes, and Vmono and Vbi represent the mean volumes of mononucleated and binucleated cardiomyocytes, respectively. Morphometric analysis was performed by investigators blinded to developmental stage or treatment group.

### Estimated ventricular cardiomyocyte number

We employed an estimated ventricular cardiomyocyte number (eVCN) as a proxy for relative cardiomyocyte abundance, based on the methodological principle that the quotient of total cardiac tissue volume and mean cell volume yields an estimate of cardiomyocyte number in neonatal heart (Li et al., 1996; Gerdes et al., 1986). The ventricular tissue volume was calculated from the ventricular weight (in μg) divided by the tissue-specific gravity (1.06) and converted to cubic micrometers (μm^3^). This total volume was then divided by the weighted average cardiomyocyte volume (in μm^3^) to generate the index for each heart. The eVCN was then derived using the following formula: eVCN=ventricular tissue volume (μm^3^)/weighted average cardiomyocyte volume (μm^3^). Statistical comparisons of group means were conducted to evaluate differences across developmental stages and between treatment groups at specific time points, thereby tracking postnatal growth and intervention-specific effects. Morphometric analysis and cell counting were performed by investigators who were unaware of the identity of experimental groups.

### RNA-sequencing of neonatal whole-heart tissues

Whole-heart tissues were collected from neonatal male and female mice at P0.5, P2.5, P3.5 and P4.5, as well as from P3.5 hearts after ICG-001 or Ctnnb1 siRNA treatment. Three biological replicates were included for each developmental stage or treatment group, and each biological replicate consisted of one individual heart. After dissection, hearts were rapidly rinsed in ice-cold PBS, snap-frozen and used for total RNA extraction.

Poly(A) RNA sequencing was performed by BGI Genomics (Shenzhen, China) using the BGISEQ-500 platform. RNA quality was assessed using an Agilent 2100 Bioanalyzer. Poly(A)+ mRNA was enriched, fragmented, reverse-transcribed, converted into double-stranded cDNA, and used for library construction and sequencing. Raw reads were filtered using SOAPnuke v1.5.2, and clean reads were aligned to the mouse reference genome using HISAT2 v2.0.4. Gene- and transcript-level expression was quantified using Bowtie2 v2.2.5 and RSEM v1.2.12, and expression values were reported as expected counts and FPKM.

### Differential expression, enrichment and heatmap analyses

Sample quality and group separation were assessed using Pearson correlation analysis, hierarchical clustering and principal component analysis. Differential expression results generated by the BGI DEGseq pipeline were used for downstream analyses. Unless otherwise stated, differentially expressed genes were defined as genes with adjusted *P*-value<0.05 and |log2 fold change|>log2(1.5).

Volcano plots were generated using ggplot2 and ggrepel in R, with selected Wnt-, BMP-, proliferation-, maturation- and cardiomyocyte growth-associated genes annotated. GO and KEGG enrichment analyses were performed using clusterProfiler with org.Mm.eg.db, DOSE and enrichplot. Upregulated and downregulated genes were analyzed separately. GSEA was performed using fgsea and clusterProfiler, with genes ranked by log2 fold change and gene set sizes restricted to 5-500 genes.

For heatmap analysis, selected genes associated with Wnt/β-catenin signaling, BMP signaling, proliferation, cardiomyocyte maturation and cardiomyocyte growth were extracted from the RNA-seq expression matrix. FPKM values were log2-transformed as log2(FPKM+1), scaled by gene to generate row z-scores and visualized using pheatmap in R.

### Quantitative RT-PCR

RNA prepared from snap frozen heart ventricles was subject to qRT-PCR using standard techniques. Total RNA was extracted using TRIzol and reverse transcribed into cDNA employing the HiScript III RT SuperMix (Vazyme, Nanjing, China). Subsequently, mRNA expression was quantified using the ChamQ Universal SYBR qPCR Master Mix (Vazyme) on an ABI StepOne Plus Real-Time PCR system (Applied Biosystems). The data were analyzed using the comparative method (2^−ΔΔCT^), with β-actin serving as the internal control; the results were expressed relative to those in the control group. Primer sequences are listed in  Table S1.

### Western blotting

Ventricular tissues were lysed in RIPA buffer supplemented with protease and phosphatase inhibitors. Protein concentrations were determined using a BCA protein assay kit. Equal amounts of protein were separated by SDS-PAGE and transferred onto PVDF membranes. After blocking with 5% non-fat milk or BSA in TBST, membranes were incubated overnight at 4°C with the indicated primary antibodies, including antibodies against β-catenin, phospho-β-catenin (Ser33), CCND1, CCNA2, SMAD1/5/9, phospho-SMAD1 (Ser463/465)/SMAD5 (Ser463/465)/SMAD9 (Ser465/467), SMAD7 and GAPDH as the loading control. The following day, membranes were washed and incubated with HRP-conjugated secondary antibodies. Protein bands were detected using enhanced chemiluminescence and imaged using Bio-Rad ChemiDoc imaging system. Band intensities were quantified using ImageJ. Total β-catenin, CCND1, CCNA2, SMAD1/5/9 and SMAD7 levels were normalized to GAPDH. Phospho-β-catenin and phospho-SMAD1/5/9 levels were normalized to total β-catenin and total SMAD1/5/9, respectively. Antibody details, including source, catalog numbers and dilutions, are provided in Table S2.

### Immunofluorescence staining of cardiac tissue sections

Cardiac tissues were harvested at P0.5, P3.5 and P4.5, and fixed in 2% paraformaldehyde. Fixed tissues were processed for paraffin embedding and sectioned at 5 μm thickness. For immunofluorescence staining, sections were deparaffinized, rehydrated, subjected to heat-induced antigen retrieval, and blocked with 10% goat serum to reduce non-specific binding. Sections were then incubated overnight at 4°C with primary antibodies. The following day, sections were washed with PBS and incubated with species-appropriate secondary antibodies, including goat anti-rabbit Alexa Fluor 488 and goat anti-mouse Alexa Fluor 594. Nuclei were counterstained with DAPI. Whole-heart sections were imaged using an Olympus VS200 whole-slide scanner. For colocalization and semi-quantitative fluorescence intensity analyses, regions of interest were randomly selected from different areas of each cardiac section. For comparisons within the same staining experiment, images were acquired using identical laser power, exposure, gain and offset settings. β-Catenin localization in cardiomyocytes was evaluated by co-staining with cTnT, and fluorescence overlap within cTnT-positive regions was analyzed using ImageJ. Antibody details, including source, catalog numbers and dilutions, are provided in Table S2.

### Combined EdU and PCM1 immunofluorescence double staining

For EdU labeling, mice were subcutaneously injected with EdU (50 mg/kg; Beyotime Biotechnology, C0078S, Shanghai, China) at P1.5 and P2.5. Cardiac tissues were harvested at P3.5, fixed, processed for paraffin embedding and sectioned at 5 μm thickness. Sections were deparaffinized, rehydrated and subjected to heat-induced antigen retrieval. EdU was then detected using a Click reaction solution prepared according to the manufacturer's instructions; sections were incubated for 30 min at room temperature. After EdU detection, sections were blocked with 10% goat serum, followed by overnight incubation at 4°C with anti-PCM1 primary antibody (see Table S2 for details). After washing, sections were incubated with the appropriate secondary antibody (e.g. goat anti-rabbit Alexa Fluor 488). Nuclei were counterstained with DAPI. Quantitative images were acquired using an Olympus VS200 whole-slide scanner with a 40× objective under identical settings and analyzed using ImageJ. Representative high-resolution images in Fig. 6L were acquired separately using an Olympus SpinSR spinning-disk confocal microscope with a 60× objective to visualize the perinuclear PCM1 pattern. Image acquisition settings for comparisons were kept identical as described above. The percentage of PCM1-positive cells incorporating EdU was quantified using ImageJ.

### Public single-cell RNA-seq analysis

Publicly available mouse cardiac single-cell RNA-seq datasets were obtained from the Gene Expression Omnibus, including GSE230531 and GSE232466. These datasets cover embryonic and early postnatal stages of mouse heart development, including E8.5, E10.5, E12.5, E14.5, E16.5, P1, P2, P3, P4 and P6. All downstream analyses were performed in R using Seurat. All samples were first processed with CellBender to correct background signals in the raw single-cell count matrices, and the corrected expression matrices were subsequently imported into Seurat for downstream analysis. Low-quality cells and potential doublets were removed based on sample-level quality-control metrics and scDblFinder. Data normalization and variance stabilization were then performed using SCTransform, followed by dimensionality reduction, dataset integration with Harmony, UMAP visualization, graph-based clustering and cell-type annotation based on canonical marker genes.

After quality control and integration, a total of 100,159 cells were retained, with 67,094 cells from GSE230531 and 33,065 cells from GSE232466. Major cardiac cell populations were annotated according to established marker genes, including cardiomyocytes, endothelial cells, epicardial cells, fibroblast-like mesenchymal cells, pericyte/smooth muscle cells, macrophages, T cells, early neural/glial progenitor-like cells, mesothelial-like epithelial cells and erythroid cells.

After global annotation, 33,858 cells were initially annotated as cardiomyocytes. Given that the E8.5 and E10.5 samples were derived from embryo trunks after removal of the head and limbs, and that early embryonic cellular states differed substantially from later cardiac developmental stages, downstream cardiomyocyte state analysis focused on the E12.5 to P6 stages. After excluding E8.5 and E10.5, 33,337 cardiomyocyte-like cells were used for re-dimensionality reduction and re-clustering. Re-clustering yielded 16 clusters, among which cluster 15 contained 201 cells and showed marker features inconsistent with typical cardiomyocytes, together with non-cardiomyocyte-associated signals. This cluster was therefore excluded, leaving 33,136 clean cardiomyocytes for subsequent developmental state annotation, cell composition analysis and pseudotime analysis.

To assess cardiomyocyte proliferation, maturation, growth-associated remodeling and signaling pathway activity, cell-cycle phase assignment and developmental module scoring were performed. Predefined developmental and pathway-related gene sets were used to evaluate the activity of Wnt/β-catenin, canonical and noncanonical Wnt, BMP and other developmental programs at the single-cell level. Cardiomyocyte developmental progression was inferred using Slingshot based on UMAP embeddings and cardiomyocyte state annotations, and changes in pathway module scores were visualized along the pseudotime trajectory.

To determine whether Wnt- and BMP-associated programs identified in bulk whole-heart RNA-seq were reflected in specific cardiac cell populations, Wnt and BMP pathway gene expression was further examined across major annotated cell types and developmental stages. For each gene, the percentage of expressing cells and the average expression level were calculated, and expression dynamics were visualized after within-cell-type scaling.

### Statistical analysis

Sample sizes were determined based on previous studies (Naqvi et al., 2014; Li et al., 1996) and no formal power calculation was performed. No animals were excluded from the analysis. Animals were randomly assigned to experimental groups using a random number table. Statistical analyses were performed using GraphPad Prism version 9.0 and R. Data distribution was assessed before statistical testing. For normally distributed data, values are presented as mean±s.e.m. Comparisons between two groups were performed using unpaired two-tailed Student's *t*-test, and comparisons among multiple groups were performed using one-way or two-way ANOVA followed by Šídák's multiple comparisons test or Fisher's least significant difference test, as appropriate. For non-normally distributed data, values are presented as median with interquartile range (IQR). Comparisons between the two groups were performed using the Mann–Whitney *U* test, and comparisons among multiple groups were performed using the Kruskal–Wallis test followed by Dunn's multiple comparisons test. Unless otherwise stated, biological replicates refer to independent animals or independent cardiomyocyte preparations. For cell-level measurements, such as cardiomyocyte length, width, volume and nucleation status, values were first averaged within each animal, and the animal-level mean was used as one biological replicate for statistical analysis. *P*<0.05 was considered statistically significant.

## Supplementary Material



10.1242/develop.205448_sup1 Supplementary information
