# Supplementary material for: Postnatal heart development is coordinated by multi-lineage Wnt and BMP signaling
Source: Development. 2026 Oct 6;153(19):dev205448. doi: 10.1242/dev.205448 (PMC13723415; doi:10.1242/dev.205448)
Supplement: Supplementary file 1 [file develop-153-205448-s1.pdf]

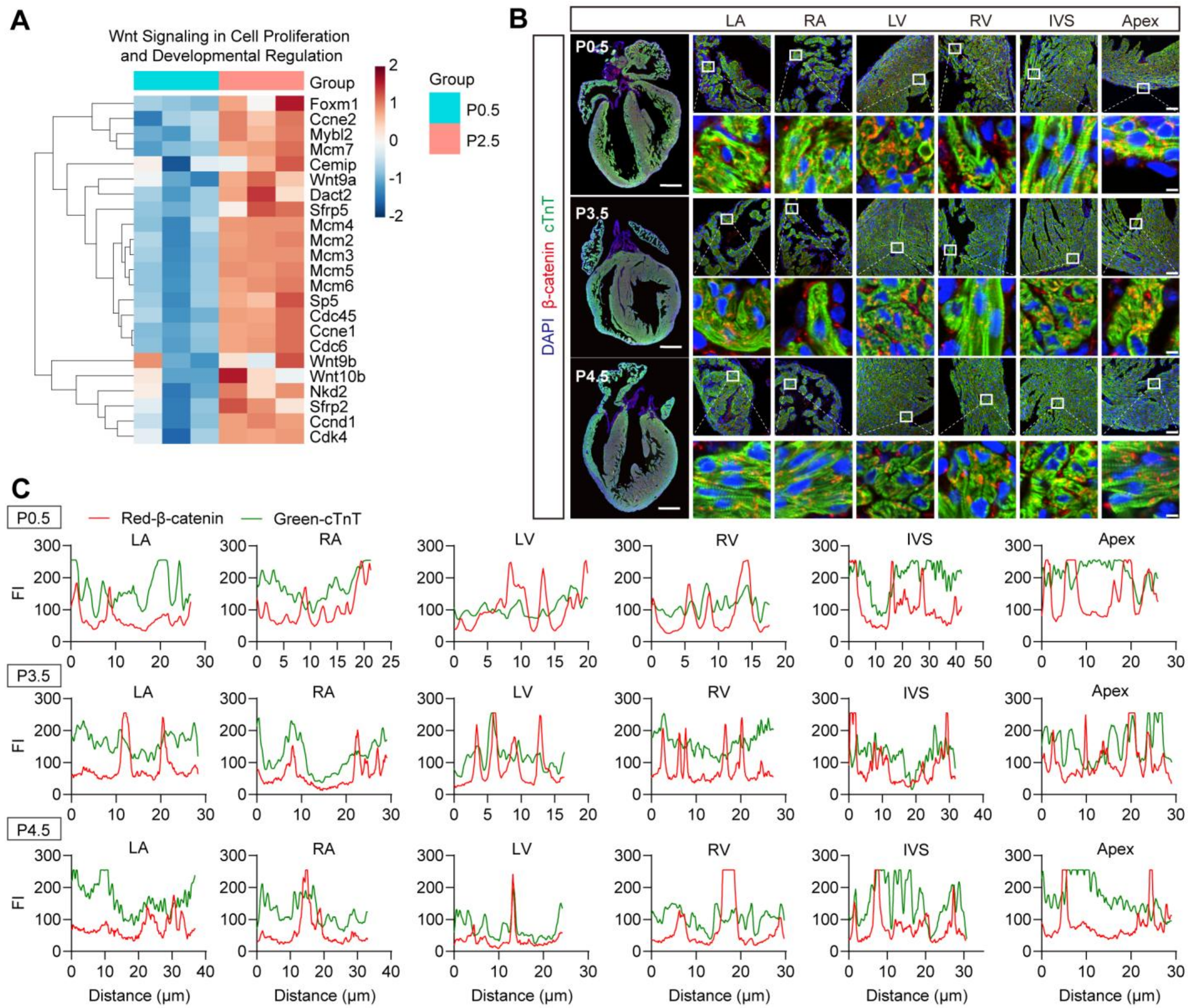

Fig. S1. Wnt-associated gene activation and β-catenin localization during early postnatal heart development

(A) Heatmap showing the expression patterns of selected Wnt-associated and cell-cycle-related genes in whole-heart transcriptomes from P0.5 and P2.5 neonatal mice. P2.5 hearts showed increased expression of several Wnt-associated genes, including *Wnt10b, Wnt9a, Wnt9b, Sp5, Nkd2, Sfrp2, Sfrp5*, and *Dact2*, together with increased expression of cell-cycle and DNA replication-related genes, including *Ccnd1, Ccne1, Cdk4, Foxm1, Mybl2, Mcm2–Mcm7, Cdc6,* and *Cdc45.* These data suggest that the P0.5-to-P2.5 transition is associated with coordinated changes in Wnt-related and cell-cycle-associated transcriptional programs. (B)

Development • Supplementary information

Representative immunofluorescence images of β-catenin and cTnT in neonatal hearts at P0.5, P3.5 and P4.5. Regional images are shown for the left atrium (LA), right atrium (RA), left ventricle (LV), right ventricle (RV), interventricular septum (IVS) and cardiac apex. DAPI was used to label nuclei. Scale bars: 0.5 mm for whole-heart images, 50 μm for regional images and 5 μm for magnified insets. (C) Representative fluorescence intensity profiles of β-catenin and cTnT signals across selected myocardial regions at P0.5, P3.5 and P4.5. Profiles are shown for LA, RA, LV, RV, IVS and cardiac apex to assess the spatial overlap between β-catenin and cTnT signals. FI, fluorescence intensity.

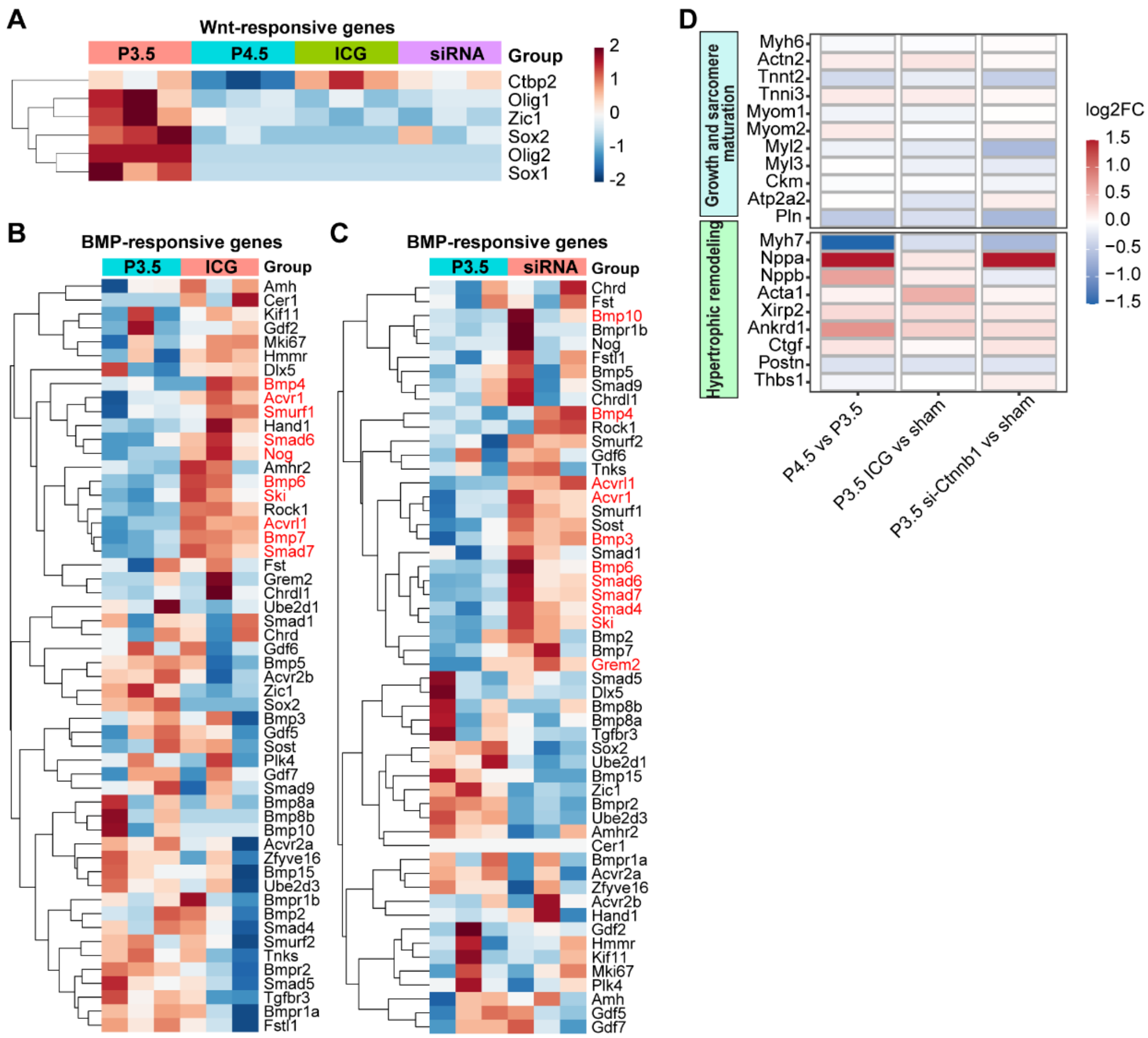

Fig. S2. ICG-001 and Ctnnb1 knockdown partially recapitulate the P3.5-to-P4.5 transcriptional remodeling of Wnt-responsive, BMP-responsive, and cardiac growth-related genes.

(A) Heatmap showing the expression patterns of a P3.5-enriched Wnt-responsive and development-associated gene subset, including Ctbp2, Zic1, Sox2, Olig2, Olig1, and Sox1, across P3.5, P4.5, ICG-001-treated, and Ctnnb1 siRNA-treated hearts. Both ICG-001 treatment and Ctnnb1 knockdown were associated with reduced expression of this gene subset, resembling the developmental decline observed from P3.5 to P4.5. (B, C) Heatmaps showing the expression patterns of BMP-responsive genes in P3.5 hearts after ICG-001 treatment (B) or Ctnnb1 siRNA treatment (C), compared with corresponding control hearts. (D) Heatmap showing log2 fold changes of representative genes associated with cardiomyocyte growth, sarcomere organization, and cardiac remodeling. Comparisons include P4.5 versus P3.5, P3.5 ICG-001-treated hearts versus P3.5 sham controls, and P3.5 Ctnnb1 siRNA-treated hearts versus P3.5 negative control siRNA-treated hearts.

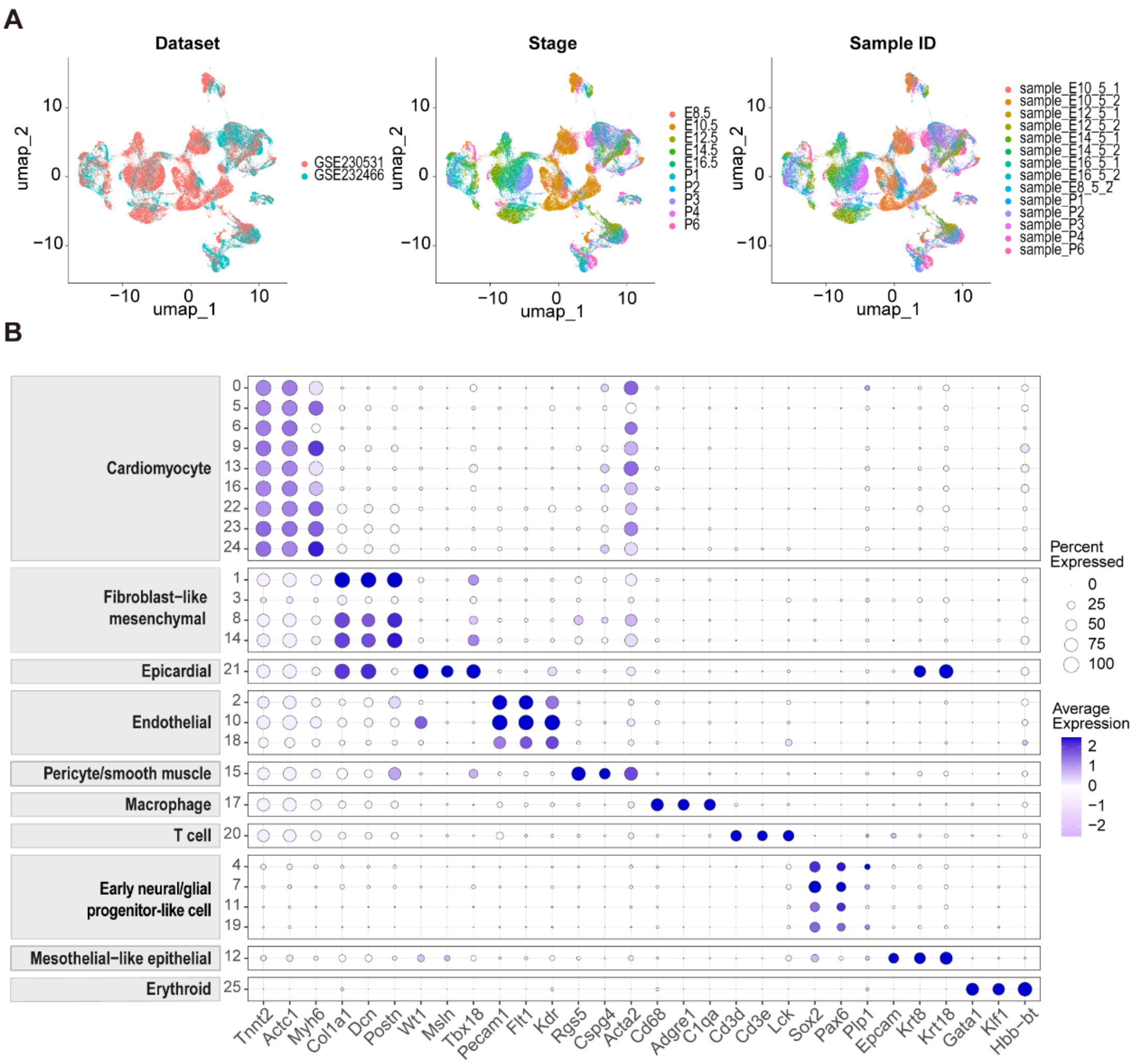

Fig. S3. Integration and major cell-type annotation of public mouse cardiac single-cell RNA-seq datasets.

(A) UMAP plots of the integrated single-cell RNA-seq dataset colored by dataset, developmental stage, and sample identity. Samples from GSE230531 and GSE232466 spanning embryonic and early postnatal stages were included. (B) Dot plot showing representative marker gene expression across original clusters and annotated major cardiac cell types. Dot size represents the percentage of expressing cells, and color intensity indicates scaled average expression. These annotations were used for the major cell-type map in Fig. 7A.

Development • Supplementary information

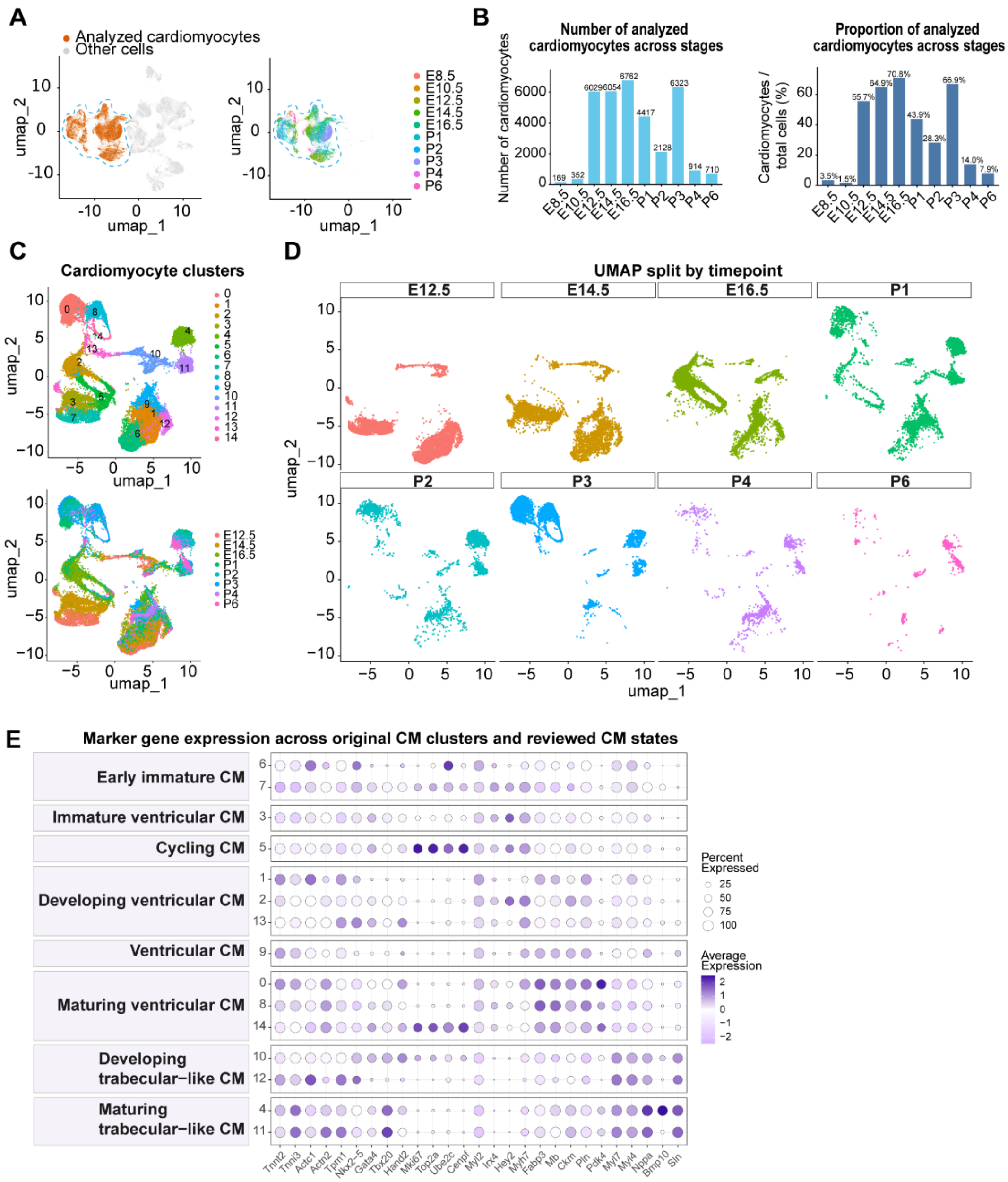

Fig. S4. Extraction, reclustering, and developmental state annotation of cardiomyocytes from the integrated public mouse cardiac single-cell RNA-seq dataset.

(A) UMAP visualization showing the cardiomyocytes selected for downstream analysis from the integrated cardiac single-cell RNA-seq dataset. Analyzed cardiomyocytes are highlighted in orange, and all other cells are shown in gray. The right panel shows the distribution of analyzed cardiomyocytes across developmental stages. (B) Number and proportion of analyzed cardiomyocytes across developmental stages. (C) UMAP visualization of reclustered cardiomyocytes, colored by cardiomyocyte subcluster and developmental stage. (D) UMAP visualization of reclustered cardiomyocytes split by developmental stage from E12.5 to P6. (E) Dot plot showing the expression of representative marker genes across cardiomyocyte subclusters and their corresponding annotated cardiomyocyte developmental states.

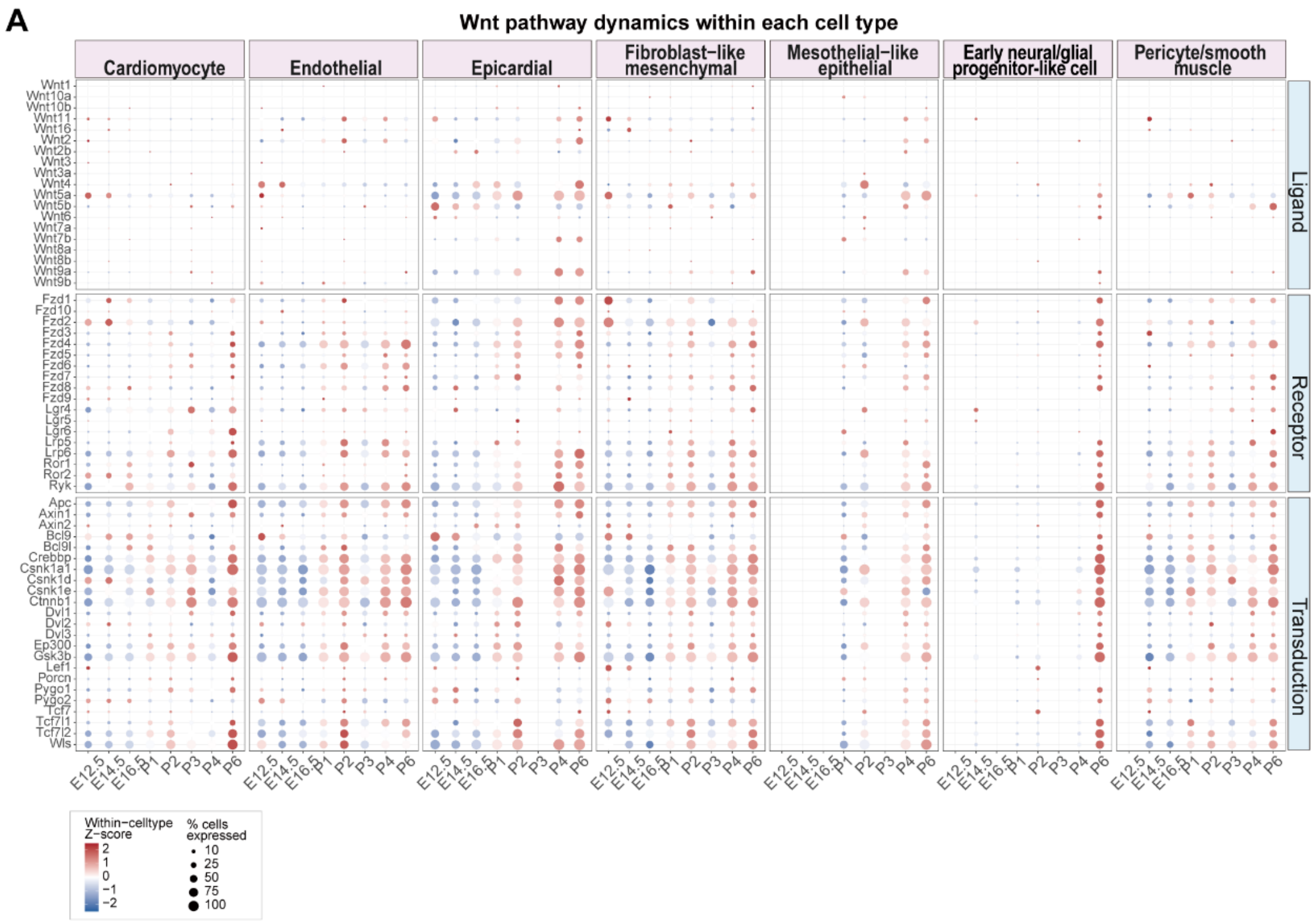

Fig. S5. Cell-type-specific dynamics of Wnt pathway gene expression during embryonic-to-postnatal heart development.

Dot plot showing the expression dynamics of Wnt pathway-associated genes across major cardiac cell types and developmental stages from E12.5 to P6. Genes were grouped into ligand, receptor, and intracellular transduction categories. Cell types included cardiomyocytes, endothelial cells, epicardial cells, fibroblast-like mesenchymal cells, mesothelial-like epithelial cells, early neural and glial progenitor-like cells, and pericyte and smooth muscle cells. Dot size represents the percentage of cells expressing each gene, and color indicates the within-cell-type scaled average expression level.

Development • Supplementary information

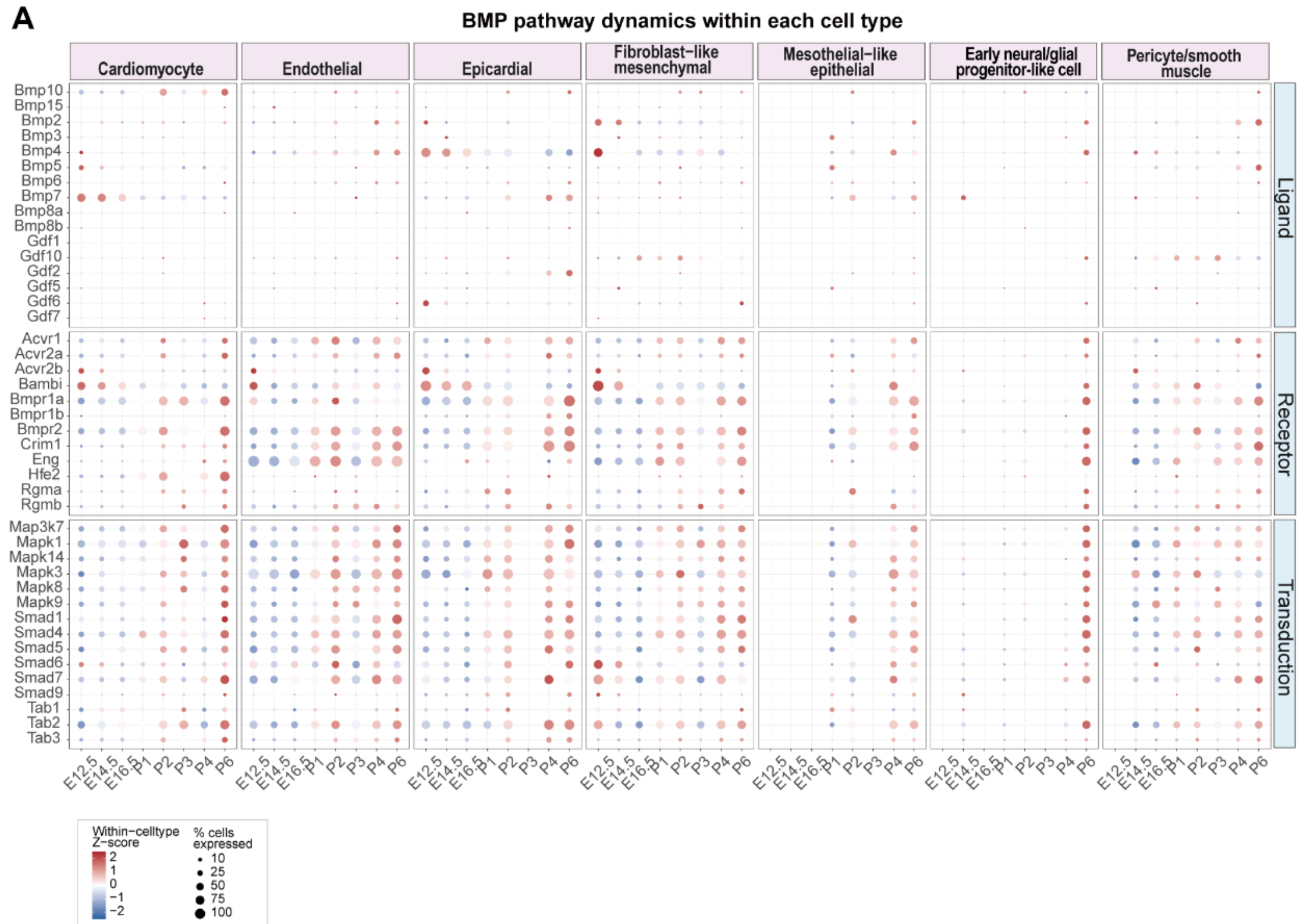

Fig. S6. Cell-type-specific dynamics of BMP pathway gene expression during embryonic-to-postnatal heart development.

Dot plot showing the expression dynamics of BMP pathway-associated genes across major cardiac cell types and developmental stages from E12.5 to P6. Genes were grouped into ligand, receptor, and intracellular transduction categories. Cell types included cardiomyocytes, endothelial cells, epicardial cells, fibroblast-like mesenchymal cells, mesothelial-like epithelial cells, early neural and glial progenitor-like cells, and pericyte and smooth muscle cells. Dot size represents the percentage of cells expressing each gene, and color indicates the within-cell-type scaled average expression level.

Table S1. PCR primers

Target Genes	Forward Primer (5′ to 3′)	Reverse Primer (5′ to 3′)
Ctnnb1	TCAGCTCGTGTCCTGTGAAG	GACCCTCTGAGCCCTAGTCA
Dkk1	CAGTGCCACCTTGAACTCAGT	CCGCCCTCATAGAGAACTCC
Dkk2	ACCCGCTGCAATAATGGAATC	ATGGTTGCGATCTCTATGCCG
Dkk3	CAGCTCTCAACTACCCTCAGG	ACCTCAGAGGACGTTTTAGCA
Sost	AGCCTTCAGGAATGATGCCAC	CTTTGGCGTCATAGGGATGGT
Apc2	CATTTGACGAAGAATACCGTCGG	CGGGTCATCTTGTGCATTTCA
Axin1	GGCCACGAGACCATGAAGG	GAAGAGCGAGCACAGGAACT
Ccnd1	CATTTGACGAAGAATACCGTCGG	CGGGTCATCTTGTGCATTTCA
β-actin	CTAAGGCCAACCGTGAAAG	GGTACGACCAGAGGCATACA

Table S2. Antibody information

Antibodies	Source	Identifier	Dilution
for immunofluorescence			
Cardiac Troponin T Polyclonal Antibody	Proteintech	15513-1-AP	1:200
Beta Catenin Monoclonal Antibody	Proteintech	66379-1-Ig	1:200
PCM1 Polyclonal Antibody	Proteintech	19856-1-AP	1:200
CoraLite488-conjugated Goat Anti-Rabbit IgG	Proteintech	SA00013-2	1:200
CoraLite594-conjugated Goat Anti-Mouse IgG	Proteintech	SA00013-3	1:200
for western blot			
Beta Catenin Monoclonal Antibody	Proteintech	66379-1-Ig	1:5000
Phospho-β- Catenin (Ser33) monoclonal Antibody	Proteintech	80067-1-RR	1:1000
Cyclin D1 (CCND1) Polyclonal Antibody	Affinity	AF0931	1:1000
Cyclin A2 (CCNA2) Polyclonal Antibody	Proteintech	18202-1-AP	1:1000
SMAD1/5/9 Polyclonal Antibody	Affinity	AF0614	1:1000
Phospho-SMAD1 (Ser463/465)/ SMAD5 (Ser463/465)/ SMAD9 (Ser465/467) (D5B10) Rabbit Monoclonal Antibody	CST	13820	1:1000
SMAD7 Polyclonal Antibody	Affinity	AF5147	1:1000
GAPDH (D4C6R) Mouse Monoclonal Antibody	CST	97166	1:1000

Development • Supplementary information
