# Supplementary material for: Postnatal heart development is coordinated by multi-lineage Wnt and BMP signaling
Source: Development. 2026 Oct 6;153(19):dev205448. doi: 10.1242/dev.205448 (PMC13723415; doi:10.1242/dev.205448)
Supplement: Supplementary file 2 [file develop-153-205448_review_history.pdf]

Postnatal heart development is coordinated by multi-lineage Wnt and BMP signaling

Hui An, Hao Hu, Saijing Chen, Tingting Chen, Yafei Hu, Tongtong Yu, Anlan Wang, Shengwei Jin and Ming Li
DOI: 10.1242/dev.205448

Editor: Benoit G. Bruneau

Review timeline

Original submission:	15 December 2025
Editorial decision:	26 February 2026
First revision received:	22 June 2026
Editorial decision:	6 July 2026
Second revision received:	25 August 2026
Accepted:	27 August 2026

Original submission

First decision letter

MS ID#: dev.205448

MS TITLE: A postnatal cardiomyocyte fate switch coordinated through coupling of declining Wnt/ß-catenin signaling with a Ctbp2-Sox2-Zic1-Olig2 module

AUTHORS: Hui An; Hao Hu; Saijing Chen; Yafei Hu; Tongtong Yu; Anlan Wang; Shengwei Jin and Ming Li

Dear Dr An,

I have now received all the referees' reports on the above manuscript, and have reached a decision. The referees' comments are appended below.

As you will see, the referees express considerable interest in your work, but have some significant criticisms and recommend a substantial revision of your manuscript before we can consider publication. One reviewer in particular questioned conclusions and claims about the effects of Wnt signaling and BMP induced differentiation from P3.5 to P4.5 and its effects on hypertrophy. If you are able to revise the manuscript along the lines suggested, which may involve further experiments, I will be happy to receive a revised version of the manuscript. Your revised paper will be re-reviewed by one or more of the original referees, and acceptance of your manuscript will depend on your addressing satisfactorily the reviewers' major concerns. Please also note that Development will normally permit only one round of major revision. If it would be helpful, you are welcome to contact us to discuss your revision in greater detail. Please send us a point-by-point response indicating your plans for addressing the referees' comments, and we will look over this and provide further guidance.

Please attend to all of the reviewers' comments and ensure that you upload both a 'clean' version of your Word file, along with a highlighted version clearly showing where you have made changes in the revised manuscript. Please avoid using 'Tracked changes' in Word files as these are lost in PDF conversion. I should be grateful if you would also provide a point-by-point response detailing how

© 2026. Published by The Company of Biologists under the terms of the Creative Commons Attribution License (https://creativecommons.org/licenses/by/4.0/).

you have dealt with the points raised by the reviewers in the 'Response to Reviewers' box. If you do not agree with any of their criticisms or suggestions please explain clearly why this is so.

Reviewer 1

This manuscript provides a strong and informative analysis of early postnatal cardiomyocyte development, focusing on phenotypic and transcriptomic changes observed between postnatal day 0.5 and 4.5. The authors identified key genes involved in the activation of the Wnt signaling pathway during the early neonatal period and illustrates how the developmental program shifts from a proliferative state toward cardiomyocyte maturation through activation of the BMP pathway by P4.5. The major strength of the study is its use of transcriptomic data to directly capture these narrow temporal changes in gene expression, allowing for a detailed and timed view of pathway activation across these early postnatal ages. There are areas where it could be further strengthened, such as: (1) while the authors imply that hypertrophic growth accompanies this developmental transition, the evidence is limited to cardiomyocyte size measurements (length and volume). The manuscript would benefit from explicitly identifying and analyzing pro-hypertrophic genes and/or demonstrating whether their activation occurs downstream of BMP signaling, (2) the paper would benefit by adding the activated genes in their schematic, (3) organization of figure citations needs to be fixed, as several figures are referenced out of order/not referenced, which disrupts the logical flow of the results. (4) Lastly, minor revisions to wording throughout the manuscript would improve clarity and readability.

Specific comments:
Line 115 - The authors need to clarify how maturation is achieved and what features does a CM becomes mature/initiates hypertrophy.
Line 173 - How is this a "contained/limiting" activation? The authors discuss this later in discussion, but feels open to interpretation and not meaningful in this space. Maybe leave this for the discussion only?
Line 215 - The authors explain Wnt and BMP shifts as bursts, which is confusing.
Line 243 - Gene Sost seems upregulated in both P0.5 and 4.5 (Figure 3B) - They need to explain.
Lines 272 to 275 - Authors did not cite Figure 5, then cite Figure 5O and start to cite Figure 5 in later point.
Line 278 - Authors introduce dataset on siRNA treatment (Figure 5O) without explaining the treatment performed.
Line 286 - Even though authors show the shift for BMP signaling pathway activation, I believe showing genes associated with hypertrophic growth would be beneficial - instead of saying that it would LIKELY be a regulator.
Line 302 - The hypertrophy is only demonstrated by CM length and volume measurements - but not in RNA/transcript data, which is not strong enough to make such a statement.
Line 317 - Suggestion: add a set of BMP genes to this image for easy comparison between groups ICG and siRNA (Figure 5O) - it is only showing expression of key proliferation-associated genes (including CtBP2, Zic1, Sox2, Olig2, Olig1 and Sox1) under the two treatments for p3.5 and p4.5.
Line 348 - Was this the only gene upregulated after CHIR administration?? -> the authors treat CMs with CHIR and they only cite this gene to be upregulated by the treatment. How is this treatment successful?
Line 404 - Which genes are activated and which are downregulated?
Line 446 - authors need to be careful with the affirmation that hypertrophy state is achieved after PDS with such early postnatal datapoints and only length and volume measurement.
Line 496 - Recommendation: Using their RNA dataset from P0.5 to 4.5 -> create heatmap with genes that are associated with hypertrophy in cardiomyocytes: such as HDAC5, mir-15 family, Myh6, CDKN1A (p21), MBNL1...
Line 850 - Legend states Phalloidin and Dapi but figure only shows phalloidin (Figure 1)
Line 884 - Small figure- needs to be enlarged for better reading - or at least the colored legends need to be bigger (Figure 2A)
There are many typos and sentences that need adjustments for better clarification. -e.g. I strongly recommend removing the "natural" or "naturally" when referring to untreated samples.

Reviewer 2

SUMMARY OF THE ADVANCE MADE IN THIS PAPER AND ITS POTENTIAL SIGNIFICANCE TO THE FIELD

© 2026. Published by The Company of Biologists under the terms of the Creative Commons Attribution License (https://creativecommons.org/licenses/by/4.0/).

This research study evaluates a cardiovascular neonatal gene expression system dependent on beta-catenin signaling and a Proliferation Differentiation Switch (PDS) module. A coordinated developmental program is required for neonatal cardiomyocyte plasticity and cell proliferation and transitioning to differentiation. Differentiation is marked by a suppression of Wnt/beta-catenin signaling and the activation of a BMP signaling axis. This signaling induces hypertrophic growth. The researchers provide several lines of investigative evidence including biological, bioinformatics and pharmaceutical studies in mice. While the findings are novel and potentially significant for the field, several key experimental validations and methodological clarifications are necessary before the results can be definitively interpreted for publication.

SUGGESTIONS TO AUTHORS
Comments:

Fig. 1. It is interesting that the authors chose to focus on P3.5 to P4.5 as a transition, whereas cardiomyocytes continue to proliferate (some reports up to 2 weeks post-natal), while shifting to hypertrophic growth. This raises the question of analyzing later neonate stages such as P7.5-P12.5, to observe an effect. Do the authors have previous data on these time points?
Concern: Manuscript must leverage public scRNA-seq data to better define proposed pathway.
The use of single-cell/single-nuclei RNA-sequencing data is critical given the proposed cell-specific signaling axis.
Public Data Integration: Include and leverage publicly available scRNA-seq datasets from neonatal mouse hearts to track the expression of key genes (e.g., Ctnnb2, Ctbp2, Sox2, and BMP pathway factors) from embryonic stages through P21, ensuring the findings are contextualized within established developmental timelines.
Cell Type Specificity: Clarify which cell population(s) were used for generating volcano plots and all subsequent scRNA-seq analyses. Not clear if only cardiomyocytes were analyzed or if the entire heart cell population was included.
Trajectory Analysis: Utilize advanced bioinformatic pipelines such as Monocle, Slingshot, and/or RNA velocity to robustly solidify claims regarding developmental trajectory and the proposed signaling axis, particularly as it relates to the proliferation-to-maturation shift (Fig 3).

Fig. 3. The authors appear to consider a subset of genes to make their point about differential expression of Wnt/beta-catenin genes vs Bmp related genes. Fig. 3 A shows an increase in Lgr5, Wnt10b, Wnt5b, Wnt3a, Wnt9a and frizzled receptors at P4.5. It appears that some Wnt signaling is not decreased at P4.5, can the authors add more explanation as to their model concerning Wnt signaling down-regulation at P4.5. Are only specific Wnt regulators decreased and are these specific for cardiomyocytes? Are Wnt inhibitors that are decreased specific for cardiomyocytes? It would be beneficial to show protein expression for these factors as 0.5-fold decrease in transcripts may not reflect functional protein concentration differences.
Interestingly, Noggin is increased at P4.5 inhibiting Bmp signaling and permitting cell proliferation. Please explain how this fits with the hypothesis. Again, for these factors, protein expression differences must be confirmed.
Fig. 4. Must show that beta-catenin is in fact decreased by Western blot. How did the CM nucleus (%) change after treatment compared to no treatment? The DM % was less than 20% in normal mice at P3.5. Also, inhibition of Wnt according to the hypothesis should activate BMP signaling leading to differentiation at P3.5. Also without undergoing terminal differentiation it is unclear how mononucleated cells undergo hypertrophy, why do they not fully differentiate, because the BMP program was activated. Did the researchers show that BMP was decreased after treatment (protein)? Thus, why the specific effect of hypertrophic growth in mononucleated cardiomyocytes when BMP is up regulated (Fig. S2A) and not terminal differentiation?
Fig. 5. Must show decreased beta-catenin protein by Western blot. Would be nice to show genes that are not affected in panel O associated with proliferation. See comments for Fig. 4, However, is this figure CM Nucleus % is shown, and there is no difference, which is surprising since the BMP pathway is activated. The differences in Fig. 4 and 5 results need to be reconciled. To make conclusions about the lack of BMP induced cardiomyocyte differentiation (when Wnt/beta-catenin expression is decreased) that an experiment where BMP signaling is activated is required. What happens to P3.5-P4.5 cardiomyocytes when BMP is activated (which is what reduced Wnt signaling is apparently doing to the cells.). If beta-catenin acts as a master regulatory node and its suppression induces the BMP program (lines 319-325) then differentiation should proceed on

© 2026. Published by The Company of Biologists under the terms of the Creative Commons Attribution License (https://creativecommons.org/licenses/by/4.0/).

mononucleated cells and subsequent hypertrophy. Does BMP activation override this master regulation?
Concern: Lack of convincing evidence that the pharmacological and genetic manipulations of the Wnt/ß-Catenin pathway reflect changes in cardiomyocyte biology.
The current evidence relies heavily on whole-heart or bulk tissue measurements following intervention. Direct cardiomyocyte-specific evidence is required.
In Vivo Expression and Localization: Provide immunocytochemistry (ICC) co-staining for Ctnnb2, Ctbp2, and Sox2 with a definitive cardiomyocyte marker (e.g., Tnnt2, Myh6/7, Nkx2.5, or PCM1) in the neonatal heart (P0-P4). This is essential to confirm that the factors are indeed expressed dynamically within cardiomyocytes.
Ex Vivo Cardiomyocyte Experiments: Perform pharmacological and genetic manipulations of Ctnnb2 on isolated primary neonatal cardiomyocytes. Assess resulting changes in:
— Gene expression of proposed targets.
— Proliferation rate (via EdU/BrdU incorporation and Ki67/PCNA ICC staining).
— Maturation markers (e.g., multinucleation, myocyte size).
Cardiomyocyte Purity: Detail the purity of primary cardiomyocyte isolations, ideally referencing a standardized FACS protocol (e.g., Waas et al, PMID: 30686762) to ensure the observed effects are genuinely cardiomyocyte intrinsic.

Fig. 6. Must show increased Wnt/beta-catenin protein using the GSK-3 inhibitor. It is not surprising that increased beta-catenin promotes proliferation. Does this further decrease BMP signaling? Are the genes that activate BMP signaling decreased? How is the PDS module affected?
Lines 408-412 are without experimental validation. The authors did not discover Olig2 as a transcription factor in heart development that acts in concert with Sox2 and Zic1. No data presented reveals a novel and sophisticated regulatory module controlling cardiomyocyte fate with these factors. These statements must be removed or validated.
Overall Concerns: Lack of methodology and experimental details provided within the manuscript. Key experimental details are missing, hindering the reviewer's ability to fully assess the rigor of the study.
RNA-seq Source: Explicitly state whether the RNA-seq data presented in the manuscript (especially Figure 1 and related analyses) was generated from whole hearts or from an enriched cardiomyocyte population.
Figure 1A Clarification: Explain the meaning of the open versus closed circles in Figure 1A and provide a clear explanation for the large variance observed in the P0.5 samples.
qPCR Source: Specify the source of RNA used for all qPCR data (Whole hearts? Isolated cardiomyocytes?).
Imaging Documentation: Provide representative ICC images alongside all quantitative data for multi-nucleation and myocyte size.
Proliferation Quantification: When quantifying EdU or general cardiomyocyte proliferation, authors must co-stain EdU with a CM nuclear marker (e.g., Nkx2.5 or PCM1) to ensure accurate approximation of cardiomyocyte-specific proliferation rates.

The manuscript tackles an important topic and uses sophisticated techniques to propose a novel regulatory axis. While the hypothesis is compelling, the current data lack the necessary cell-type specificity and methodological clarity to fully support the conclusions. Addressing the concerns outlined above, particularly using public scRNA-seq data and rigorous validation in isolated primary cardiomyocytes, will significantly enhance the manuscript's impact and interpretability.

First revision

<u>Author response to reviewers' comments</u>

Revised title: Postnatal heart development is coordinated by multi-lineage Wnt and BMP signaling

Original title: A postnatal cardiomyocyte fate switch coordinated through coupling of declining Wnt/ß-catenin signaling with a Ctbp2-Sox2-Zic1-Olig2 module

© 2026. Published by The Company of Biologists under the terms of the Creative Commons Attribution License (https://creativecommons.org/licenses/by/4.0/).

Dear Editor and Reviewers,

We sincerely thank the Editor and reviewers for their careful evaluation of our manuscript and for their constructive suggestions. We have substantially revised the manuscript to better align the conclusions with the data, to improve methodological transparency, and to provide additional transcriptomic, protein-level, imaging, and single-cell analyses.

Because final line numbers may shift during journal formatting and highlighting, we refer below to the revised manuscript by section and figure/panel.

Summary of major revisions

Revision area	Summary
Central model and terminology	We revised the title, abstract, introduction, results and discussion to avoid overinterpretation. Terms such as "fate switch," "terminal differentiation," "PDS collapse" and "Wnt-to-BMP relay" have been removed or substantially toned down. The revised text describes an early postnatal transition from cell-cycle-associated activity toward cardiomyocyte enlargement and growth-associated remodeling.
Growth-associated transcriptional programs	We added analyses of representative cardiac growth- and remodeling-associated genes and compared their expression across the P3.5-to-P4.5 developmental transition, ICG-001 treatment and Ctnnb1 knockdown (Fig. S2D).
Protein-level validation	We added or expanded western blot analyses for β-catenin, degradation-associated phospho-β-catenin Ser33, CCND1, CCNA2, phospho-SMAD1/5/9, total SMAD1/5/9 and SMAD7 where appropriate (Figs. 3E,F; 4N,O; 5B,C; 6O).
Perturbation experiments	We reorganized the ICG-001, Ctnnb1 siRNA and CHIR-99021 results so each intervention is introduced before the corresponding data are presented (Figs. 4-6).
Single-cell RNA-seq	We integrated public mouse cardiac single-cell RNA-seq datasets (GSE230531 and GSE232466) to resolve cell-type context, cardiomyocyte developmental states, module scores, Slingshot pseudotime and Wnt/BMP pathway dynamics (Fig. 7; Figs. S3-S6).
Sample source and cell-type specificity	We clarified which data were generated from whole-heart or ventricular tissue, isolated cardiomyocytes, all single cells, or reclustered cardiomyocytes.
Figures, legends and Methods	We revised figure legends, improved figure readability, clarified biological replicates and statistics, and added antibody details to Supplementary Materials and Methods and Table S1.

Response to Reviewer 1

We thank Reviewer 1 for recognizing the strength of the transcriptomic analysis and for the constructive suggestions that helped improve the rigor, organization and clarity of the manuscript.

1.1. Evidence for cardiomyocyte growth and BMP downstream programs

> ***Reviewer comment:*** *"While the authors imply that hypertrophic growth accompanies this developmental transition, the evidence is limited to cardiomyocyte size measurements. The manuscript would benefit from explicitly identifying and analyzing pro-hypertrophic genes and/or demonstrating whether their activation occurs downstream of BMP signaling."*

© 2026. Published by The Company of Biologists under the terms of the Creative Commons Attribution License (https://creativecommons.org/licenses/by/4.0/).

Response: We thank the reviewer for this important comment. We agree that the original wording overstated the evidence for "hypertrophic growth," because the conclusion was based primarily on cardiomyocyte morphometry. We have revised the terminology throughout the manuscript and now refer to cardiomyocyte enlargement, growth-associated remodeling, or muscle growth-associated transcriptional programs, rather than claiming that a fully established hypertrophic state is achieved.

To address the reviewer's suggestion, we added an analysis of representative cardiac growth- and remodeling-associated genes across the P3.5-to-P4.5 developmental transition, ICG-001 treatment, and *Ctnnb1* knockdown (Fig. S2D). This analysis showed partial overlap between the developmental P3.5-to-P4.5 transition and the transcriptional changes induced by β-catenin/CBP disruption or *Ctnnb1* knockdown. We also added BMP-responsive gene analyses after ICG-001 treatment and *Ctnnb1* knockdown (Fig. S2B,C). At the protein level, ICG-001 increased phospho- SMAD1/5/9 relative to total SMAD1/5/9 and reduced SMAD7 protein levels (Fig. 4N,O), supporting BMP/SMAD-related signaling remodeling in this context.

We further used single-cell RNA-seq to place BMP pathway genes in their cellular context (Fig. S6). Our whole-heart RNA-seq data showed stage-dependent regulation of Bmp4 and Bmp10, and the single-cell analysis suggested distinct cellular patterns for these ligands: Bmp10 was enriched in cardiomyocytes, whereas Bmp4 showed stronger expression in non-cardiomyocyte populations, including endothelial and fibroblast-like mesenchymal cells. In parallel, cardiomyocytes expressed BMP receptors and downstream SMAD-related components, including Bmpr1a, Bmpr2, Smad1, Smad4, Smad5, and Smad7, particularly at later postnatal stages in the integrated single-cell dataset (Fig. S6). These observations are consistent with the possibility that BMP-related signaling during early postnatal development involves communication between non-cardiomyocyte populations and cardiomyocytes.

This interpretation is supported by previous studies showing that BMP signaling can regulate cardiomyocyte growth-related responses. BMP4 has been reported to activate SMAD1/5/8 signaling and induce hypertrophy-associated markers such as ANP and BNP in cardiomyocyte models [1,2]. In addition, postnatal myocardial Bmp10 overexpression was reported to impair normal postnatal cardiac growth, with reduced heart size attributed to defective postnatal hypertrophic growth [3]. We therefore added these references to support the biological relevance of BMP-related signaling to cardiomyocyte growth.
Importantly, we have also revised the manuscript to avoid stating that BMP activation alone directly drives cardiomyocyte enlargement downstream of Wnt/β-catenin inhibition. Because we did not perform BMP gain-of-function, BMP inhibition, or ligand-source-specific perturbation experiments, we now conclude more conservatively that β-catenin attenuation is accompanied by BMP/SMAD-related signaling changes and growth-associated transcriptional remodeling.

Changes made:

- Replaced overstrong terms such as "hypertrophic growth," "terminal differentiation," and "BMP-driven hypertrophy" with "cardiomyocyte enlargement" and "growth-associated remodeling" where appropriate.
- Added cardiac growth- and remodeling-associated gene analysis in **Fig. S2D**.
- Added BMP-responsive gene analyses in **Fig. S2B,C**.
- Added protein-level evidence for BMP/SMAD-related signaling changes in **Fig. 4N,O**.
- Added cell-type-resolved BMP pathway analysis in **Fig. S6**.
- Revised the Results and Discussion to state that BMP/SMAD-related remodeling is associated with, rather than proven to directly drive, cardiomyocyte enlargement.

1.2. Activated genes in the schematic/model and gene-level clarity

Reviewer comment: *"The paper would benefit by adding the activated genes in their schematic."*

Response: We thank the reviewer for this constructive suggestion. We agree that a schematic summarizing key gene programs would be beneficial in principle. However, during revision we

© 2026. Published by The Company of Biologists under the terms of the Creative Commons Attribution License (https://creativecommons.org/licenses/by/4.0/).

re-evaluated our working model and realized that the original schematic risked overinterpreting the data by implying a simple linear pathway and by not adequately representing cellular heterogeneity (for example, the Sox/Zic/Olig-containing module was mainly enriched in non-cardiomyocyte populations, as shown in Fig. 7B). To avoid overstating the conclusions, we decided to remove the original schematic entirely from the revised manuscript.

Instead of a simplified model figure, we now provide gene-level information in a data-driven manner using two complementary approaches: Bulk RNA-seq results (Fig. S2B–D) show BMP-responsive gene remodeling after ICG-001 and *Ctnnb1* knockdown, as well as representative cardiac growth- and remodeling-associated genes across the P3.5-to-P4.5 transition and perturbation conditions. Single-cell RNA-seq results (Fig. S5, S6) provide cell-type-resolved expression patterns of Wnt and BMP pathway genes, avoiding the misleading impression that all changes are cardiomyocyte-intrinsic.

We believe that presenting these empirical data, rather than a potentially oversimplified schematic, better supports our conclusions and aligns with the overall revision goal of avoiding overinterpretation. If the Editor nevertheless feels that a minimal schematic is necessary for clarity, we would be happy to prepare a very simplified version that lists representative genes while clearly stating its limitations. This point has also been noted in the revised Discussion.
Changes made:

- Removed the original model schematic from the revised manuscript.
- Added bulk RNA-seq gene-level heatmaps in Fig. S2B–D.
- Added single-cell RNA-seq cell-type-resolved pathway analyses in Fig. S5 and S6.

1.3. Figure citation order and logical flow

Reviewer comment: "Organization of figure citations needs to be fixed, as several figures are referenced out of order/not referenced, which disrupts the logical flow of the results."

Response: We thank the reviewer for pointing this out. We have carefully re-examined the entire Results section and reorganized the order in which figures and panels are cited. Each figure is now introduced before its individual panels are described. Specifically, we restructured the Ctnnb1 knockdown subsection to present the validation (Fig. 5A–C) before the phenotypic analyses (Fig. 5D–P), and we moved the perturbation-associated transcriptional heatmaps to Fig. S2 to avoid interrupting the main flow. We believe the revised Results section now follows a logical, sequential order.

1.4. Wording, typos and readability

Reviewer comment: "Lastly, minor revisions to wording throughout the manuscript would improve clarity and readability."

Response: We agree. We have thoroughly proofread the entire manuscript, corrected typos, simplified overly complex sentences, and replaced imprecise terms (e.g., removed "natural/naturally" when referring to untreated samples, as also suggested by the reviewer). We also standardized terminology for developmental stages and experimental conditions. These changes have been made throughout the main text, figure legends, and Methods. We believe the revised manuscript is substantially clearer and more readable.

1.5. Clarification of cardiomyocyte maturation and hypertrophic growth terminology

Reviewer comment: *"Line 115 - The authors need to clarify how maturation is achieved and what features does a CM becomes mature/initiates hypertrophy."*

Response: We agree that the original text did not sufficiently distinguish cardiomyocyte maturation, physiological enlargement and hypertrophic growth. We have revised the Introduction and Results to clarify that cardiomyocyte maturation is a broader process involving coordinated structural, metabolic, electrophysiological, proliferative and molecular changes, whereas our experimental measurements mainly support cardiomyocyte enlargement, binucleation and growth-associated remodeling during the early postnatal period. We therefore avoid using "maturation" or "terminal differentiation" as the primary conclusion for Fig. 1 and instead describe the observed process as an early postnatal transition involving cardiomyocyte enlargement and growth-associated remodeling.

© 2026. Published by The Company of Biologists under the terms of the Creative Commons Attribution License (https://creativecommons.org/licenses/by/4.0/).

Changes made:

- Clarified cardiomyocyte maturation terminology in the Introduction.
- Revised Fig. 1 and Fig. 2 interpretations to avoid equating enlargement with complete maturation.
- Reduced use of "terminal differentiation" throughout the manuscript.

1.6. Overinterpretation of "contained/limiting activation"

> ***Reviewer comment:*** *"Line 173 - How is this a 'contained/limiting' activation? ... Maybe leave this for the discussion only?"*

Response: We agree. The phrase "contained/limiting activation" was interpretive and not necessary in the Results. We removed this wording from the Results and replaced it with descriptive terms such as stage-dependent regulation, dynamic remodeling and transient enrichment. Where a broader interpretation is useful, it has been moved to the Discussion and framed as a possible interpretation rather than a conclusion.

Changes made:

- Removed "contained/limiting activation" from the Results.
- Revised corresponding text to use descriptive language.
- Moved speculative interpretation to the Discussion and toned down the wording.

1.7. Use of "bursts" to describe Wnt and BMP shifts

> ***Reviewer comment:*** *"Line 215 - The authors explain Wnt and BMP shifts as bursts, which is confusing."*

Response: We agree that "burst" is imprecise and could imply a stronger and more uniform pathway activation than supported by the data. We replaced this term with more accurate descriptions, including transient increase, stage-dependent remodeling, dynamic regulation and reciprocal signaling pattern, depending on the context. We also revised the Wnt/BMP model to avoid implying a simple on/off switch.

Changes made:

- Removed "burst" terminology.
- Replaced it with "stage-dependent remodeling," "dynamic regulation," or "transient increase" where appropriate.
- Revised Wnt/BMP-related interpretations to avoid overstatement.

1.8. Sost expression pattern

> ***Reviewer comment:*** *"Line 243 - Gene Sost seems upregulated in both P0.5 and 4.5 (Figure 3B) - They need to explain."*

Response: We thank the reviewer for this careful observation. Indeed, *Sost* mRNA appears relatively high at both P0.5 and P4.5 in our dataset (Fig. 3B). We agree that this finding requires clarification, particularly because *Sost* is not typically considered a cardiomyocyte-enriched gene.

Existing evidence indicates that cardiac Sost expression during the perinatal period can arise from non-cardiomyocyte lineages. Van der Plas et al. (2007) reported that Sost is expressed in vascular smooth muscle cells of the great arteries from embryonic day 15.5 to postnatal day 7 and is absent from the myocardium [4]. Similarly, McKean et al. (2024) showed SOST expression in cardiac endothelial cells [5]. SOST encodes sclerostin, a secreted antagonist of canonical Wnt/β-catenin signaling. Thus, the Sost signal detected in whole-heart RNA-seq at P0.5 and P4.5 may reflect contributions from developing vascular smooth muscle cells or endothelial cells, rather than cardiomyocyte-intrinsic Wnt inhibition alone.

© 2026. Published by The Company of Biologists under the terms of the Creative Commons Attribution License (https://creativecommons.org/licenses/by/4.0/).

Consistent with this interpretation, we added cell-type-resolved Wnt pathway analysis using public single-cell RNA-seq data (Fig. S5). We now describe **Sost** as part of heterogeneous, stage-dependent Wnt-related remodeling and avoid using this individual gene as evidence for global Wnt pathway suppression.

Changes made:

- Revised Results text describing Sost and Wnt modulators.
- Added cell-type-resolved Wnt pathway gene analysis in Fig. S5.
- Avoided describing Wnt signaling as globally suppressed based on individual genes.

1.9. Figure citation order and introduction of Fig. 5

Reviewer comment: *“Lines 272 to 275 - Authors did not cite Figure 5, then cite Figure 5O and start to cite Figure 5 in later point.”*

Response: We agree. We reorganized the Results so that each perturbation experiment is introduced before individual panels are cited. The Ctnnb1 knockdown experiment is now presented as a separate Results subsection, beginning with Ctnnb1 mRNA and β-catenin protein validation (Fig. 5A–C), followed by growth measurements, nuclearity, morphometry and eVCN (Fig. 5D–P). The former heatmap-style comparison has been moved to Fig. S2 to avoid interrupting the logical flow.

Changes made:

- Reordered figure citations in the Results.
- Moved perturbation-associated transcriptional heatmaps to Fig. S2.
- Ensured that Fig. 5 is introduced before specific panels are cited.

1.10. Explanation of Ctnnb1 siRNA treatment

Reviewer comment: *“Line 278 - Authors introduce dataset on siRNA treatment (Figure 5O)*

without explaining the treatment performed.”

Response: We agree that the original presentation was unclear. In the revised Results, the Ctnnb1 siRNA experiment is introduced before the data are shown. We first describe the treatment and then show validation of Ctnnb1 knockdown at the mRNA and protein levels (Fig. 5A–C). Details of siRNA administration, timing and sample collection are provided in the Methods.

Changes made:

- Added a dedicated Results subsection for Ctnnb1 knockdown.
- Added Ctnnb1 mRNA and β-catenin protein validation in Fig. 5A-C.
- Expanded siRNA treatment details in Methods.

1.11. Growth-associated genes and BMP interpretation

Reviewer comment: *“Line 286 - ... showing genes associated with hypertrophic growth would be beneficial - instead of saying that it would LIKELY be a regulator.”*

Response: This point overlaps with Comment 1.1, and we have addressed it by adding cardiac growth- and remodeling-associated gene analysis (Fig. S2D), BMP-responsive gene heatmaps (Fig. S2B,C), and protein-level SMADs and p-SMAD1/5/9 readouts (Fig. 4O). We also revised the text to avoid stating that BMP is a direct regulator of cardiomyocyte hypertrophy in our system. The revised manuscript states that BMP/SMAD-related remodeling accompanies cardiomyocyte enlargement and growth-associated transcriptional changes.

Changes made:

- Added Fig. S2B–D and Fig. 4O.
- Revised causal language regarding BMP signaling.
- Replaced “likely regulator” wording with more data-specific phrasing.

© 2026. Published by The Company of Biologists under the terms of the Creative Commons Attribution License (https://creativecommons.org/licenses/by/4.0/).

1.12. Hypertrophy claim based only on cell size

> ***Reviewer comment:*** *"Line 302 - The hypertrophy is only demonstrated by CM length and volume measurements - but not in RNA/transcript data, which is not strong enough to make such a statement."*

Response: We agree. We have revised the manuscript to avoid claiming that a complete hypertrophic state is achieved solely on the basis of length and volume measurements. We now use cardiomyocyte enlargement and growth-associated remodeling as the main descriptive terms. The new Fig. S2D provides complementary transcriptional analysis of cardiac growth- and remodeling-associated genes, but we still interpret these findings conservatively and do not claim full hypertrophic maturation.

Changes made:

- Replaced "hypertrophy" with "cardiomyocyte enlargement" where appropriate.
- Added Fig. S2D to provide transcriptional support for growth-associated remodeling.
- Revised Discussion to emphasize limitations of morphometry-only interpretations.

1.13. Add BMP genes for comparison between ICG-001 and Ctnnb1 siRNA

> ***Reviewer comment:*** *"Line 317 - Suggestion: add a set of BMP genes to this image for easy comparison between groups ICG and siRNA."*

Response: We appreciate this suggestion. Rather than overcrowding the original panel, we reorganized the transcriptional comparison into Fig. S2. Fig. S2B shows BMP-responsive genes after ICG-001 treatment, Fig. S2C shows BMP-responsive genes after Ctnnb1 knockdown, and Fig. S2D shows representative cardiac growth- and remodeling-associated genes across the P3.5- to-P4.5 transition and both perturbation conditions.

Changes made:

- Added BMP-responsive gene heatmaps in Fig. S2B,C.
- Added cardiac growth/remodeling-associated gene heatmap in Fig. S2D.
- Moved the original perturbation transcriptional comparison to the supplementary figure set for clarity.

1.14. CHIR-99021 treatment efficacy

> ***Reviewer comment:*** *"Line 348 - Was this the only gene upregulated after CHIR administration? ... How is this treatment successful?"*

Response: We agree that Ccnd1 mRNA alone was insufficient to demonstrate CHIR efficacy. We therefore added protein-level and imaging evidence. CHIR reduced ß-catenin phosphorylation at Ser33, a degradation-associated site, consistent with ß-catenin stabilization (Fig. 6A). CHIR also increased Cyclin D1 and Cyclin A2 protein levels (Fig. 6O), increased EDU-positive PCM1-positive cardiomyocytes (Fig. 6L,M), and increased estimated ventricular cardiomyocyte number (Fig. 6K). These data together support successful activation of ß-catenin-associated cell-cycle output.

Changes made:

- Added CHIR western blot validation in Fig. 6A.
- Added/clarified EdU/PCM1 data in Fig. 6L–M.
- Revised Results to present CHIR efficacy using multiple readouts rather than Ccnd1 alone.

1.15. Specify activated and downregulated genes

> ***Reviewer comment:*** *"Line 404 - Which genes are activated and which are downregulated?"*

Response: We revised the Results and Discussion to specify gene programs rather than using vague activated/downregulated terminology. The P0.5-to-P2.5 interval is described as enriched for DNA replication, mitotic cell-cycle, E2F targets and G2M checkpoint programs (Fig. 2C,D; Fig. S1). The P3.5-to-P4.5 interval is described as showing upregulation of muscle system process, regulation of

© 2026. Published by The Company of Biologists under the terms of the Creative Commons Attribution License (https://creativecommons.org/licenses/by/4.0/).

developmental growth, extracellular matrix organization and regulation of cell growth, with downregulation of DNA replication and cell-cycle-related processes (Fig. 2F–H). We also explicitly describe the Sox/Zic/Olig-containing developmental gene subset, BMP-responsive genes, and cardiac growth/remodeling-associated genes in Fig. S2.

Changes made:

- Revised Results text to name gene categories and representative gene sets.
- Added Fig. S1 and Fig. S2 references where relevant.
- Removed vague “activated/downregulated genes” statements.

1.16. Caution regarding hypertrophy state after PDS

Reviewer comment: *“Line 446 - authors need to be careful with the affirmation that hypertrophy state is achieved after PDS with such early postnatal datapoints and only length and volume measurement.”*

Response: We agree and have substantially revised this part of the manuscript. We no longer describe the Sox/Zic/Olig-containing module as a cardiomyocyte-intrinsic PDS that drives a hypertrophy state. Instead, single-cell analysis shows that this module is enriched primarily in early neural and glial-like cells (Fig. 7B). We now state that the P3.5-to-P4.5 interval is associated with cardiomyocyte enlargement, binucleation and growth-associated remodeling, rather than a definitive hypertrophic state following a PDS.

Changes made:

- Removed or toned down PDS and cardiomyocyte fate-switch language.
- Revised Discussion to avoid claiming that a hypertrophy state is achieved after PDS.
- Added Fig. 7B to clarify the cellular context of the Sox/Zic/Olig-containing module.

1.17. Heatmap of hypertrophy-associated genes

Reviewer comment: *“Line 496 - Recommendation: Using their RNA dataset from P0.5 to 4.5 create heatmap with genes that are associated with hypertrophy in cardiomyocytes, such as HDAC5, miR-15 family, Myh6, CDKN1A (p21), MBNL1...”*

Response: We thank the reviewer for the helpful suggestion. We added a heatmap of representative cardiac growth- and remodeling-associated mRNA genes (Fig. S2D). Because our dataset is mRNA-seq, miRNA family members such as the miR-15 family could not be directly analyzed. However, we included detectable mRNA genes associated with cardiac growth, remodeling and structural programs, and we interpret these data as evidence of growth-associated remodeling rather than complete hypertrophic maturation.

Changes made:

- Added representative cardiac growth- and remodeling-associated gene heatmap in Fig. S2D.
- Clarified that miRNA species could not be directly assessed from the mRNA-seq dataset.
- Revised terminology to avoid overstating hypertrophy.

1.18. Figure 1 legend and DAPI display

Reviewer comment: *“Line 850 - Legend states Phalloidin and DAPI but figure only shows phalloidin (Figure 1).”*

Response: We thank the reviewer for noting this discrepancy. We revised Fig. 1F and its legend to ensure that the displayed image channels and legend match. The revised legend now accurately states the staining used to visualize cardiomyocyte boundaries and nuclei.

Changes made:

- Revised Fig. 1F and legend.
- Checked figure legends to ensure displayed channels match the descriptions.

© 2026. Published by The Company of Biologists under the terms of the Creative Commons Attribution License (https://creativecommons.org/licenses/by/4.0/).

1.19. Figure 2A readability

Reviewer comment: "Line 884 - Small figure needs to be enlarged for better reading, or at least the colored legends need to be bigger (Figure 2A)."

Response: We agree. We enlarged Fig. 2A and increased the font size of the labels and legend to improve readability.

Changes made:

- Enlarged Fig. 2A.
- Increased PCA legend and label font sizes.

1.20. Wording, typos and removal of "natural/naturally"

Reviewer comment: "There are many typos and sentences that need adjustments for better clarification. I strongly recommend removing 'natural' or 'naturally' when referring to untreated samples."

Response: We agree. We edited the manuscript throughout for grammar, clarity and readability. We removed unnecessary uses of "natural" or "naturally" when referring to untreated or developmental samples and replaced them with more precise terms such as developmental transition, physiological postnatal development or vehicle-treated controls where appropriate.

Changes made:

- Proofread and revised the manuscript throughout.
- Removed "natural/naturally" where used imprecisely.
- Standardized terminology for untreated, vehicle-treated and developmental conditions.

Response to Reviewer 2

We thank Reviewer 2 for the thoughtful and constructive evaluation of our manuscript. We appreciate the reviewer's recognition that the study addresses an important question in neonatal cardiac development. The reviewer raised several important concerns regarding timing, Wnt/BMP interpretation, cell-type specificity, cardiomyocyte-specific evidence and methodological clarity.

For clarity, we respond to the reviewer's comments in the order in which they were raised, with multipart comments divided into numbered subpoints.

2.1. Rationale for focusing on P3.5-to-P4.5

Reviewer comment: "Fig. 1. It is interesting that the authors chose to focus on P3.5 to P4.5 as a transition, whereas cardiomyocytes continue to proliferate ... Do the authors have previous data on P7.5-P12.5?"

Response: We thank the reviewer for raising this important point. We do not intend to claim that all postnatal cardiomyocyte proliferation ends at P4.5. Rather, our study focuses on the very early postnatal growth transition identified by our own measurements. In Fig. 1, HW/BW and VW/BW show a transient elevation around P4.5 (Fig. 1A–E), cardiomyocyte volume increases most prominently between P3.5 and P4.5 (Fig. 1N,O), double-nucleated cardiomyocytes increase from P3.5 onward (Fig. 1G), and estimated ventricular cardiomyocyte number remains relatively stable after P2.5 (Fig. 1P). Whole-heart RNA-seq further shows that P3.5-to-P4.5 is the interval in which cell-cycle-associated transcriptional programs decline while muscle growth-associated programs increase (Fig. 2F–H). We therefore focus on P3.5-to-P4.5 as an early postnatal transition window, not as the endpoint of all neonatal cardiomyocyte proliferative capacity. We do not currently have systematic morphometric and perturbation data at P7.5–P12.5, and we have revised the manuscript to restrict our conclusions to the early postnatal period analyzed here.

© 2026. Published by The Company of Biologists under the terms of the Creative Commons Attribution License (https://creativecommons.org/licenses/by/4.0/).

Changes made:

- Clarified that the study focuses on the early P3.5-to-P4.5 transition rather than the entire neonatal proliferative period.
- Revised language to avoid implying that cardiomyocyte proliferation fully ceases at P4.5.
- Added/clarified this limitation in the Discussion.

2.2. Integration of public single-cell RNA-seq datasets

Reviewer comment: *"Manuscript must leverage public scRNA-seq data to better define proposed pathway... Include publicly available scRNA-seq datasets from neonatal mouse hearts to track key genes."*

Response: We agree that cell-type-resolved analysis is essential for interpreting whole-heart transcriptomic data. We therefore integrated two public mouse cardiac single-cell RNA-seq datasets, GSE230531 and GSE232466, spanning embryonic and early postnatal stages. After quality control, doublet removal, normalization and integration, 100,159 cells were retained for analysis. We annotated major cardiac cell populations (Fig. 7A; Fig. S3), then extracted and reclustered 33,136 high-confidence cardiomyocytes from E12.5 to P6 for developmental state analysis (Fig. 7C–J; Fig. S4). We also analyzed Wnt and BMP pathway gene expression across major cardiac cell types (Figs. S5,S6). Although the reviewer suggested extending to P21, the datasets that met our integration and quality criteria in this revision covered E8.5 to P6. We therefore limit the single-cell conclusions to the embryonic-to-early-postnatal window and no longer extrapolate beyond this period.

Changes made:

- Added integrated public scRNA-seq analysis in Fig. 7 and Figs. S3–S6.
- Added major cell-type annotation, cardiomyocyte reclustering, module scoring and Slingshot pseudotime analysis.
- Restricted conclusions to the developmental stages covered by the integrated datasets.

2.3. Clarification of cell populations used for each analysis

Reviewer comment: *"Cell Type Specificity: Clarify which cell population(s) were used for generating volcano plots and all subsequent scRNA-seq analyses. Not clear if only cardiomyocytes were analyzed or if the entire heart cell population was included."*

Response: We agree that this distinction must be explicit. In the revised manuscript, we state that the volcano plots, GO and GSEA analyses in Fig. 2 and the Wnt/BMP heatmaps in Fig. 3 were generated from whole-heart bulk RNA-seq. qPCR and western blot experiments were performed using tissue samples as specified in the Methods. In contrast, cardiomyocyte nuclearity and morphometry were quantified using isolated ventricular cardiomyocytes. For the single-cell analysis, Fig. 7A,B and Fig. S3 use all integrated cardiac cells; Fig. 7C–J and Fig. S4 use extracted and reclustered cardiomyocytes; and Figs. S5,S6 show pathway gene expression across major annotated cardiac cell types.

Changes made:

- Clarified sample source and cell population for each analysis in Results, Methods and figure legends.
- Explicitly distinguished whole-heart transcriptomic data from cardiomyocyte morphometry and single-cell analyses.

2.4. Trajectory analysis

Reviewer comment: *"Trajectory Analysis: Utilize advanced bioinformatic pipelines such as Monocle, Slingshot, and/or RNA velocity to solidify claims regarding developmental trajectory..."*

Response: We agree and have added Slingshot pseudotime analysis of reclustered cardiomyocytes. Fig. 7H shows developmental and signaling module scores ordered along pseudotime, while Fig.

© 2026. Published by The Company of Biologists under the terms of the Creative Commons Attribution License (https://creativecommons.org/licenses/by/4.0/).

7I,J show smoothed canonical Wnt and BMP signaling scores along the trajectory. Proliferation-associated modules, including G1/S, G2/M and cardiomyocyte proliferation scores, were enriched at early pseudotime and declined progressively. Sarcomere maturation, calcium handling, metabolic maturation, physiological hypertrophy and ventricular maturation scores increased toward intermediate-to-late pseudotime. Wnt inhibitor scores and BMP signaling scores increased in late pseudotime regions. We revised the text to describe this as a transition from cell-cycle-associated programs toward structural and growth-associated cardiomyocyte programs.

Changes made:

- Added Slingshot pseudotime analysis in Fig. 7H–J.
- Revised Results to describe ordered pseudotime dynamics of proliferation, structural, metabolic, Wnt-inhibitory and BMP-related programs.

2.5. Interpretation of Wnt signaling at P4.5

Reviewer comment: *“Fig. 3A shows an increase in Lgr5, Wnt10b, Wnt5b, Wnt3a, Wnt9a and frizzled receptors at P4.5. It appears that some Wnt signaling is not decreased at P4.5...”*

Response: We agree that the original model was too simple. We no longer describe Wnt signaling as globally decreased or turned off at P4.5. Instead, the revised manuscript distinguishes between declining β-catenin abundance at the protein level (Fig. 3E,F), dynamic expression of Wnt pathway-associated genes in whole-heart RNA-seq (Fig. 3A), and cell-type-dependent Wnt gene expression in single-cell RNA-seq (Fig. S5). Thus, some Wnt ligands and receptors can increase at P4.5 while β-catenin abundance declines and Wnt-related programs show heterogeneous regulation across cell types. This revised interpretation avoids a global Wnt-off model.

Changes made:

- Revised Results and Discussion to describe Wnt-related remodeling rather than global Wnt suppression.
- Added cell-type-resolved Wnt pathway analysis in Fig. S5.
- Clarified that β-catenin protein abundance, Wnt ligand expression and Wnt inhibitor expression are distinct readouts.

2.6. Protein-level support for Wnt/BMP changes

Reviewer comment: *“It would be beneficial to show protein expression for these factors as 0.5- fold decrease in transcripts may not reflect functional protein concentration differences.”*

Response: We agree. We added or expanded protein-level analyses. During development, β-catenin protein abundance decreases from P0.5 to P3.5/P4.5, whereas pSMAD1/5/9 relative to total SMAD1/5/9 increases (Fig. 3E,F). After ICG-001 treatment, degradation-associated pβ-catenin Ser33 increases, Cyclin D1 decreases, pSMAD1/5/9 increases and SMAD7 decreases (Fig. 4N,O). Ctnnb1 siRNA reduces β-catenin protein abundance (Fig. 5B,C). CHIR-99021 reduces degradation-associated pβ-catenin Ser33 and increases Cyclin D1 and Cyclin A2 (Fig. 6O,P). We also added β-catenin/cTnT immunofluorescence to provide spatial myocardial context (Fig. 3G,H).

Changes made:

- Added/expanded western blots in Figs. 3E,F; 4N,O; 5B,C; 6A.
- Added β-catenin/cTnT immunofluorescence and line-scan analysis in Fig. 3G,H.
- Clarified phospho-β-catenin Ser33 as a degradation-associated site in figure legends and Results.

2.7. Noggin and BMP pathway interpretation

Reviewer comment: *“Noggin is increased at P4.5 inhibiting Bmp signaling and permitting cell proliferation. Please explain how this fits with the hypothesis.”*

© 2026. Published by The Company of Biologists under the terms of the Creative Commons Attribution License (https://creativecommons.org/licenses/by/4.0/).

Response: We thank the reviewer for this comment. We agree that the increase in Noggin indicates that BMP pathway regulation is not unidirectional. We have therefore revised the manuscript to avoid a simple “BMP-dominant” or on/off model. We now describe BMP/SMAD-related remodeling as a pathway state that includes ligands, receptors, intracellular SMAD components and feedback regulators. An increase in a BMP antagonist such as Noggin can be interpreted as part of feedback regulation rather than evidence that BMP/SMAD signaling is absent. Consistent with this, pSMAD1/5/9 increases at the protein level during early postnatal development (Fig. 3E,F).

Changes made:

- Revised Wnt/BMP interpretation to emphasize heterogeneous and feedback-regulated pathway remodeling.
- Removed or toned down simple BMP-dominant/on-off language.
- Emphasized pSMAD1/5/9 as a protein-level readout.

2.8. ICG-001 treatment: β-catenin readout, nuclearity and BMP interpretation

> ***Reviewer comment:*** *“Fig. 4. Must show that beta-catenin is in fact decreased by Western blot. How did the CM nucleus (%) change after treatment compared to no treatment? ... why the specific effect of hypertrophic growth in mononucleated cardiomyocytes when BMP is up regulated and not terminal differentiation?”*

Response: We appreciate this detailed comment. ICG-001 disrupts β-catenin/CBP-dependent transcriptional output and is not designed to directly knock down total β-catenin protein. To better document pathway effects, we added western blot readouts showing increased β-catenin phosphorylation at Ser33, a degradation-associated site, relative to total β-catenin, together with reduced Cyclin D1 protein levels (Fig. 4N,O). These data support attenuation of β-catenin-associated cell-cycle output. Regarding nuclearity, ICG-001 did not significantly alter the proportion of single- and double-nucleated cardiomyocytes (Fig. 4F), nor did it increase estimated ventricular cardiomyocyte number (Fig. 4G). Morphometric analysis showed that ICG-001 primarily increased single-nucleated cardiomyocyte length and estimated volume (Fig. 4H–J), while double-nucleated cardiomyocyte size was not significantly changed (Fig. 4K–M). We have also revised the interpretation of BMP signaling: we no longer claim that BMP activation induces terminal differentiation in this context. Instead, ICG-001 is described as enhancing cardiomyocyte enlargement and growth-associated remodeling, accompanied by increased pSMAD1/5/9 and reduced SMAD7 (Fig. 4N,O).

Changes made:

- Added/clarified ICG-001 western blot readouts in Fig. 4N,O.
- Clarified that ICG-001 disrupts β-catenin/CBP-associated transcription rather than functioning as β-catenin knockdown.
- Clarified nuclearity results in Fig. 4F and eVCN in Fig. 4G.
- Removed terminal differentiation claims from the ICG-001 interpretation.

2.9. Ctnnb1 knockdown validation and reconciliation with ICG-001

> ***Reviewer comment:*** *“Fig. 5. Must show decreased beta-catenin protein by Western blot... The differences in Fig. 4 and 5 results need to be reconciled.”*

Response: We agree. The revised Fig. 5 now shows both Ctnnb1 mRNA reduction and β-catenin protein reduction after Ctnnb1 siRNA treatment (Fig. 5A–C). Ctnnb1 knockdown did not markedly alter cardiomyocyte nuclearity (Fig. 5I), but increased both single- and double-nucleated cardiomyocyte size (Fig. 5J–O) and reduced estimated ventricular cardiomyocyte number (Fig. 5P). We now explicitly reconcile Fig. 4 and Fig. 5: ICG-001 disrupts β-catenin/CBP-associated transcriptional output, whereas Ctnnb1 siRNA reduces β-catenin abundance more broadly. Despite these mechanistic differences, both interventions support the conclusion that β-catenin attenuation favors cardiomyocyte enlargement over cardiomyocyte number expansion during this early postnatal window.

© 2026. Published by The Company of Biologists under the terms of the Creative Commons Attribution License (https://creativecommons.org/licenses/by/4.0/).

Changes made:

- Added/clarified β-catenin protein validation in Fig. 5B,C.
- Revised Results to compare ICG-001 and Ctnnb1 siRNA outcomes.
- Clarified that the two perturbations are mechanistically related but not identical.

2.10. Need for BMP activation experiments to establish causality

Reviewer comment: *"To make conclusions about BMP induced cardiomyocyte differentiation ... an experiment where BMP signaling is activated is required."*

Response: We agree that direct BMP gain-of-function or BMP inhibition/rescue experiments would be required to establish causality. We have therefore revised the manuscript to avoid claiming that BMP activation directly induces cardiomyocyte differentiation or hypertrophy in our model. The current data support an association between β-catenin attenuation, BMP/SMAD- related remodeling and cardiomyocyte enlargement. This limitation is now acknowledged in the Discussion.

Changes made:

- Removed causal statements that BMP activation alone drives differentiation or hypertrophy.
- Revised model to state that BMP/SMAD-related remodeling accompanies the transition.
- Added limitation regarding future BMP perturbation experiments.

2.11. Cardiomyocyte-specific evidence for perturbation effects

Reviewer comment: *"The current evidence relies heavily on whole-heart or bulk tissue measurements following intervention. Direct cardiomyocyte-specific evidence is required."*

Response: We agree that this distinction is important. In the revised manuscript, we explicitly separate tissue-level molecular assays from cardiomyocyte-level analyses. Cardiomyocyte size and nuclearity were measured directly in isolated ventricular cardiomyocytes (Figs. 1, 4, 5, 6). CHIR-induced cardiomyocyte cell-cycle-associated activity was assessed using Ki67 with MYH7 and EdU with PCM1 co-staining (Fig. 6K–M). We also added β-catenin/cTnT immunofluorescence (Fig. 3G,H) and integrated single-cell RNA-seq data to resolve cardiomyocyte versus non-cardiomyocyte contributions (Fig. 7; Figs. S3–S6). Accordingly, we have revised the conclusions to avoid stating that all molecular effects are cardiomyocyte- intrinsic.

Changes made:

- Clarified tissue-level versus cardiomyocyte-level assays.
- Added EdU/PCM1 co-staining in Fig. 6L,M and β-catenin/cTnT staining in Fig. 3G,H.
- Added single-cell analysis to resolve cell-type specificity.
- Revised conclusions to avoid overclaiming cardiomyocyte-intrinsic mechanisms.

2.12. In vivo localization of β-catenin, Ctbp2 and Sox2

Reviewer comment: *"Provide immunocytochemistry co-staining for Ctnnb1, Ctbp2, and Sox2 with a definitive cardiomyocyte marker in the neonatal heart."*

Response: We thank the reviewer for this suggestion. We added β-catenin immunofluorescence with the cardiomyocyte marker cTnT and included line-scan fluorescence intensity profiles to assess spatial overlap between β-catenin and cTnT signals (Fig. 3G,H). For Ctbp2, Sox2 and related Sox/Zic/Olig genes, we addressed cellular specificity using integrated public single-cell RNA-seq. This analysis showed that the Sox/Zic/Olig-containing developmental gene subset is enriched primarily in early neural and glial-like cells, with limited expression in cardiomyocytes (Fig. 7B). Based on this result, we revised the manuscript to avoid presenting these factors as cardiomyocyte-intrinsic regulators.

Changes made:

© 2026. Published by The Company of Biologists under the terms of the Creative Commons Attribution License (https://creativecommons.org/licenses/by/4.0/).

- Added β-catenin/cTnT immunofluorescence and line-scan analysis in Fig. 3G,H.
- Added single-cell evidence for Sox/Zic/Olig cellular distribution in Fig. 7B.
- Removed claims that Ctbp2/Sox2/Zic1/Olig2 directly regulate cardiomyocyte fate.

2.13. Ex vivo cardiomyocyte experiments and cardiomyocyte purity

Reviewer comment: *"Perform pharmacological and genetic manipulations of Ctnnb1 on isolated primary neonatal cardiomyocytes... Detail the purity of primary cardiomyocyte isolations..."*

Response: We agree that ex vivo cardiomyocyte-specific perturbation experiments would be informative and would help distinguish cell-autonomous from tissue-level effects. These experiments are beyond the scope of the current revision. To address the concern within the present study, we strengthened the in vivo and cell-type-resolved evidence, including isolated cardiomyocyte morphometry, cardiomyocyte-marker co-staining for proliferation, and public single-cell RNA-seq analysis. We also expanded the Methods to describe cardiomyocyte isolation, staining, nuclearity assessment and morphometric criteria. We have revised the text to avoid claiming that all perturbation effects are strictly cardiomyocyte-autonomous.

Changes made:

- Expanded Methods for cardiomyocyte isolation and morphometry.
- Clarified that isolated cardiomyocytes were used for morphology and nuclearity quantification.
- Added limitation regarding future cell-autonomous ex vivo testing.

2.14. CHIR-99021 validation and BMP/PDS effects

Reviewer comment: *"Fig. 6. Must show increased Wnt/beta-catenin protein using the GSK-3 inhibitor... Does this further decrease BMP signaling? Are the genes that activate BMP signaling decreased? How is the PDS module affected?"*

Response: We agree that CHIR efficacy should be demonstrated using multiple readouts. We added western blot data showing that CHIR reduces β-catenin phosphorylation at Ser33, a degradation-associated site, consistent with β-catenin stabilization (Fig. 6O,P). CHIR also increased Cyclin D1 and Cyclin A2 protein levels (Fig. 6O,P), increased Ki67-positive MYH7-positive cardiomyocytes (Fig. 6K,L), increased EdU-positive PCM1-positive cardiomyocytes (Fig. 6M), and increased estimated ventricular cardiomyocyte number (Fig. 6J). We do not claim that CHIR acts through BMP suppression or PDS modulation, because those mechanisms were not directly tested in the CHIR experiment. The revised text focuses on β-catenin stabilization promoting cardiomyocyte cell-cycle-associated activity while limiting cell-volume expansion.

Changes made:

- Added CHIR western blot validation in Fig. 6A.
- Added/clarified Ki67/MYH7 and EdU/PCM1 evidence in Fig. 6L–M.
- Revised CHIR interpretation to avoid unsupported BMP/PDS claims.

2.15. Claims regarding Olig2, Sox2, Zic1 and the PDS module

Reviewer comment: *"The authors did not discover Olig2 as a transcription factor in heart development that acts in concert with Sox2 and Zic1. No data presented reveals a novel and sophisticated regulatory module controlling cardiomyocyte fate with these factors. These statements must be removed or validated."*

Response: We agree with the reviewer. The original manuscript overstated the significance of the Sox/Zic/Olig-containing gene subset and incorrectly implied that this module directly controls cardiomyocyte fate. We removed these statements and no longer present Olig2, Sox2 and Zic1 as a novel cardiomyocyte fate-regulatory module. In the revised manuscript, these genes are described more conservatively as a Sox/Zic/Olig-containing developmental gene subset. Single- cell analysis shows that this subset is enriched primarily in early neural and glial-like cells rather than cardiomyocytes (Fig. 7B), and we now interpret it as a developmental program associated with a non-myocyte cellular component of the developing heart.

© 2026. Published by The Company of Biologists under the terms of the Creative Commons Attribution License (https://creativecommons.org/licenses/by/4.0/).

Changes made:

- Removed claims that Olig2, Sox2 and Zic1 form a novel regulatory module controlling cardiomyocyte fate.
- Replaced “PDS module” and “fate switch” language with “Sox/Zic/Olig-containing developmental gene subset” where appropriate.
- Revised title, abstract, introduction, results and discussion accordingly.

2.16. RNA-seq and qPCR sample sources

> ***Reviewer comment:*** *“RNA-seq Source: Explicitly state whether the RNA-seq data ... was generated from whole hearts or from an enriched cardiomyocyte population. qPCR Source: Specify the source of RNA used for all qPCR data.”*

Response: We thank the reviewer for pointing out this omission. We have clarified the sample source for each molecular analysis. The developmental RNA-seq data in Figs. 2 and 3 were generated from whole-heart tissue. Perturbation-associated transcriptomic analyses after ICG-001 treatment or Ctnnb1 siRNA treatment were also performed using whole-heart samples. qPCR analyses were performed using ventricular tissue RNA, and western blot analyses were performed using tissue lysates as specified in the Methods. In contrast, cardiomyocyte nuclearity and morphometry were performed using isolated ventricular cardiomyocytes.

Changes made:

- Clarified RNA-seq, qPCR and western blot sample sources in Methods and figure legends.
- Clarified which analyses used whole-heart/ventricular tissue and which used isolated cardiomyocytes.

2.17. Figure 1A symbols and P0.5 variability

> ***Reviewer comment:*** *“Figure 1A Clarification: Explain the meaning of the open versus closed circles in Figure 1A and provide a clear explanation for the large variance observed in the P0.5 samples.”*

Response: We thank the reviewer for noting this issue. We revised Fig. 1A and its legend to clarify the meaning of individual data points and summary statistics. Each data point represents one biological replicate from an independent animal, and summary data are shown as mean ± SEM. We also revised the Results to avoid overinterpreting the relatively larger variability at the earliest neonatal stage, which may reflect biological variability in very small neonatal animals and the narrow timing of birth-related measurements.

Changes made:

- Revised Fig. 1A legend to clarify individual data points and summary statistics.
- Clarified biological replicate definition.
- Revised Results to avoid overinterpretation of P0.5 variability.

2.18. Imaging documentation for multinucleation and cell size

> ***Reviewer comment:*** *“Imaging Documentation: Provide representative ICC images alongside all quantitative data for multi-nucleation and myocyte size.”*

Response: We agree that representative imaging documentation is important. Fig. 1F now shows representative isolated cardiomyocytes across developmental stages used for nuclearity and morphometric analyses. The figure legend and Methods have been revised to clarify staining, imaging, and criteria for quantifying single- and double-nucleated cardiomyocytes and cell-size parameters. Where quantification was performed on isolated cardiomyocytes rather than tissue sections, we have clarified this to avoid confusion.

Changes made:

- Revised Fig. 1F and legend.

© 2026. Published by The Company of Biologists under the terms of the Creative Commons Attribution License (https://creativecommons.org/licenses/by/4.0/).

- Expanded Methods for cardiomyocyte imaging, nuclearity classification and morphometric analysis.
- Clarified that morphometry was performed on isolated ventricular cardiomyocytes.

2.19. Cardiomyocyte-specific proliferation quantification

Reviewer comment: *"When quantifying EdU or general cardiomyocyte proliferation, authors must co-stain EdU with a CM nuclear marker such as Nkx2.5 or PCM1."*

Response: We agree. We added EdU and PCM1 co-staining in CHIR-treated hearts. The revised **Fig. 6L,M** shows EdU incorporation together with PCM1 staining, and white dashed outlines indicate EdU-positive, PCM1-positive cardiomyocytes..

Changes made:

- Added EdU/PCM1 co-staining in Fig. 6L,M.
- Revised Fig. 6 legend to identify EdU-positive, PCM1-positive cardiomyocytes.
- Clarified proliferation quantification methods in Methods.

2.20. Overall methodological detail

Reviewer comment: *"Overall Concerns: Lack of methodology and experimental details provided within the manuscript. Key experimental details are missing..."*

Response: We thank the reviewer for this comment. We substantially expanded the Methods and

Supplementary Materials and Methods to improve transparency and reproducibility. We now morphometry, western blotting, immunofluorescence staining, statistical analysis, biological replicate definition, and public single-cell RNA-seq processing. Antibody details are provided in Supplementary Materials and Methods and Table S1.

Changes made:

- Expanded Methods and Supplementary Materials and Methods.
- Added antibody information in Table S1.
- Clarified biological replicate definition and statistical analyses.
- Added details of the single-cell RNA-seq pipeline, including CellBender, scDblFinder, SCTransform, Harmony, Seurat and Slingshot.

Reference

1. Wang YC, Xiao XL, Li N, Yang D, Xing Y, Huo R, et al. Oestrogen inhibits BMP4-induced BMP4 expression in cardiomyocytes: a potential mechanism of oestrogen-mediated protection against cardiac hypertrophy. Br J Pharmacol. 2015;172(23):5586-95. Epub 20150112. doi: 10.1111/bph.12983. PubMed PMID: 25323043; PubMed Central PMCID: PMCPMC4667872.

2. Sun B, Huo R, Sheng Y, Li Y, Xie X, Chen C, et al. Bone morphogenetic protein-4 mediates cardiac hypertrophy, apoptosis, and fibrosis in experimentally pathological cardiac hypertrophy. Hypertension. 2013;61(2):352-60. Epub 20121217. doi: 10.1161/HYPERTENSIONAHA.111.00562. PubMed PMID: 23248151.

3. Chen H, Yong W, Ren S, Shen W, He Y, Cox KA, et al. Overexpression of bone morphogenetic protein 10 in myocardium disrupts cardiac postnatal hypertrophic growth. J Biol Chem. 2006;281(37):27481-91. Epub 20060623. doi: 10.1074/jbc.M604818200. PubMed PMID: 16798733; PubMed Central PMCID: PMCPMC2628764.

4. van Bezooijen RL, Deruiter MC, Vilain N, Monteiro RM, Visser A, van der Wee-Pals L, et al. SOST expression is restricted to the great arteries during embryonic and neonatal cardiovascular development. Dev Dyn. 2007;236(2):606-12. doi: 10.1002/dvdy.21054. PubMed PMID: 17195180.

5. McKean DM, Zhang Q, Narayan P, Morton SU, Strohmenger V, Tang VT, et al. Increased

© 2026. Published by The Company of Biologists under the terms of the Creative Commons Attribution License (https://creativecommons.org/licenses/by/4.0/).

endothelial sclerostin caused by elevated DSCAM mediates multiple trisomy 21 phenotypes. J
PubMed Central PMCID: PMCPMC11142749.

Second decision letter

MS ID#: dev.205448R1

MS TITLE: Postnatal heart development is coordinated by multi-lineage Wnt and BMP signaling

AUTHORS: Hui An; Hao Hu; Saijing Chen; Tingting Chen; Yafei Hu; Tongtong Yu; Anlan Wang; Shengwei Jin and Ming Li

Dear Dr An,

I have now received all the referees reports on the above manuscript, and have reached a decision. The referees' comments are appended below.

The overall evaluation is positive and we would like to publish a revised manuscript in Development, provided that the referee's minor comments can be satisfactorily addressed. Please attend to all of the reviewers' comments in your revised manuscript and detail them in your point-by-point response. If you do not agree with any of their criticisms or suggestions explain clearly why this is so. If it would be helpful, you are welcome to contact us to discuss your revision in greater detail. Please send us a point-by-point response indicating your plans for addressing the referees' comments, and we will look over this and provide further guidance.

Reviewer 1

SUMMARY OF THE ADVANCE MADE IN THIS PAPER AND ITS POTENTIAL SIGNIFICANCE TO THE FIELD

The authors provided the necessary edits and implemented new data that strengthens the paper.

Overall, I am content with their response to my questions, although I would like to know points below before proceeding.

Figure 3 - The authors have added immunostaining figures from P0.5 - P4.5 and western blot results showing B-catenin levels at those time points. While the western blots show a clear decrease of B-catenin on P3.5 and P4.5, the fluorescence images seem to not be altered. Why so? Also, why on (H) only P0.5 IF intensity profile is shown?

Figure 6 - The authors have included immunofluorescence images with EdU+ and PCM1 markers, usually PCM1 is only shown in nuclear vicinity, but in those images PCM1 seems to be shown throughout the whole CM area. Is this an artifact, or the antibody used reveal the CM area?

Second revision

Author response to reviewers' comments

Dear Editor and Reviewers,

We sincerely thank the Editor and Reviewer for their careful evaluation of our revised manuscript and for the positive assessment of the additional data and analyses. We appreciate the opportunity to clarify the two remaining points concerning the apparent difference between the ß-catenin immunofluorescence and western blot results, and the PCM1 staining pattern shown in

© 2026. Published by The Company of Biologists under the terms of the Creative Commons Attribution License (https://creativecommons.org/licenses/by/4.0/).

Figure 6. We have carefully addressed these comments below and have revised the relevant text and figure information where appropriate.
Because line numbers may shift during final formatting and revision highlighting, we refer to the revised manuscript by section and figure/panel.

Response to Reviewer 1

We thank Reviewer 1 for the positive assessment of our revised manuscript and for recognizing that the additional analyses and experimental data have strengthened the study. We also appreciate the reviewer's careful evaluation of the newly added figures and the opportunity to clarify the apparent differences between the immunofluorescence and western blot results, as well as the PCM1 staining pattern.

1.1 Apparent discrepancy between β-catenin immunofluorescence and western blot results

> ***Reviewer comment:*** *"Figure 3 - The authors have added immunostaining figures from P0.5 - P4.5 and western blot results showing β-catenin levels at those time points. While the western blots show a clear decrease of β-catenin on P3.5 and P4.5, the fluorescence images seem to not be altered. Why so? Also, why on (H) only P0.5 IF intensity profile is shown?"*

Response:

We thank the reviewer for raising this important point. We agree that, in the previous version, the immunofluorescence images and western blot data could appear inconsistent. This was mainly because the original immunofluorescence panel was intended to show the spatial localization and colocalization of β-catenin with cardiomyocyte markers, rather than to provide a quantitative comparison of β-catenin abundance across developmental stages. Therefore, the apparent lack of obvious visual change in the representative IF images should not be interpreted as evidence that β-catenin levels were unchanged.

To address this concern more directly, we have now repeated the β-catenin immunofluorescence staining at P0.5, P3.5 and P4.5 and performed semi-quantitative fluorescence intensity analysis using six biological samples per group. This new analysis shows that β-catenin fluorescence intensity is reduced at P3.5 and P4.5 compared with P0.5, consistent with the decrease detected by western blotting. Thus, the revised immunofluorescence quantification and western blot analysis now support the same conclusion that β-catenin protein abundance decreases during this developmental window.
Regarding the reviewer's question about why only the P0.5 IF intensity profile was shown previously, the P0.5 profile was originally included only as an example to illustrate the colocalization pattern between β-catenin and cardiomyocyte markers. We did not intend to imply that colocalization occurred only at P0.5 or that later stages were not analyzed. In the revised manuscript, we have added the corresponding fluorescence overlap/intensity profiles for P3.5 and P4.5 to provide a consistent comparison across the examined developmental stages.

Changes made:

1. We added immunofluorescence staining and semi-quantitative fluorescence intensity analysis of β-catenin at P0.5, P3.5 and P4.5, with six biological samples per group, now shown in Fig. 3G,H.

2. We added the P3.5 and P4.5 fluorescence overlap/intensity profiles to complement the original P0.5 profile, now shown in Fig. S1B,C.

3. We revised the corresponding figure legend and Results text to clarify that the original IF images were primarily used to assess β-catenin localization/colocalization within cardiomyocyte regions, whereas quantitative IF analysis and western blotting were used to assess developmental changes in β-catenin protein abundance.

4. We revised the Methods to specify whole-heart imaging using the Olympus VS200 whole-slide scanner, random selection of regions of interest, consistent image-acquisition settings and ImageJ-based fluorescence analyses.

1.2 PCM1 immunofluorescence staining pattern in cardiomyocytes

© 2026. Published by The Company of Biologists under the terms of the Creative Commons Attribution License (https://creativecommons.org/licenses/by/4.0/).

Reviewer comment: *"Figure 6 - The authors have included immunofluorescence images with EdU+ and PCM1 markers, usually PCM1 is only shown in nuclear vicinity, but in those images PCM1 seems to be shown throughout the whole CM area. Is this an artifact, or the antibody used reveal the CM area?"*

Response:

We thank the reviewer for this important observation. PCM1 was used in this study as a marker of cardiomyocyte nuclei based on its characteristic perinuclear localization and was not used to delineate the entire cardiomyocyte area. In the original images, the PCM1 channel already showed fluorescence enrichment immediately surrounding the nuclei, with a ring- or crescent-like pattern. However, we acknowledge that the relatively high diffuse green background in the myocardial tissue reduced the contrast between the perinuclear PCM1 signal and the surrounding tissue, particularly in the merged images, and may therefore have created the impression that PCM1 staining extended throughout the cardiomyocyte area.

The original images were acquired using an Olympus VS200 whole-slide scanner with a 40× objective. To resolve this potential ambiguity more clearly, we reacquired representative images using an Olympus SpinSR spinning-disk confocal microscope with a 60× objective. The improved optical sectioning and higher spatial resolution of the confocal images more clearly demonstrate that the PCM1 signal used to identify cardiomyocyte nuclei is concentrated immediately around DAPI-positive nuclei, forming the expected ring- or crescent-like perinuclear pattern. The diffuse fluorescence within the myocardial area was not used to delineate cardiomyocyte boundaries or to determine PCM1 positivity.

We have therefore replaced the original images with the newly acquired 60× spinning-disk confocal images. To make the characteristic perinuclear localization of PCM1 more readily apparent, digitally enlarged views of the boxed regions have also been included. Yellow arrowheads indicate EdU-positive nuclei surrounded by a distinct PCM1-positive perinuclear rim. For image analysis, only DAPI-defined nuclei showing nuclear EdU incorporation together with a clear perinuclear PCM1 signal were classified as EdU-positive cardiomyocyte nuclei. These revisions improve the clarity of the presentation but do not affect the quantitative results or the conclusions of the study.

Changes made:

1. We replaced the original images in Fig. 6L with newly acquired 60× spinning-disk confocal images and added digitally enlarged views of the boxed regions to more clearly demonstrate the characteristic perinuclear localization of PCM1. We also revised the corresponding figure legend to clarify the image annotations and scale bars.

2. We revised the Methods to specify that high-resolution PCM1 images were acquired using an Olympus SpinSR spinning-disk confocal microscope with a 60× objective.

Third decision letter

MS ID#: dev.205448R2

MS TITLE: Postnatal heart development is coordinated by multi-lineage Wnt and BMP signaling

AUTHORS: Hui An; Hao Hu; Saijing Chen; Tingting Chen; Yafei Hu; Tongtong Yu; Anlan Wang; Shengwei Jin and Ming Li

Dear Dr An,

© 2026. Published by The Company of Biologists under the terms of the Creative Commons Attribution License (https://creativecommons.org/licenses/by/4.0/).

I am happy to tell you that your manuscript has been accepted for publication in Development, pending our standard publication integrity checks.

© 2026. Published by The Company of Biologists under the terms of the Creative Commons Attribution License (https://creativecommons.org/licenses/by/4.0/).
